# Distinct pathways utilized by METTL3 to regulate antiviral innate immune response

**DOI:** 10.1016/j.isci.2024.111071

**Published:** 2024-09-30

**Authors:** Haojie Hao, Fang Zhang, Zhen Chen, Zhongyuan Tan, Hongyan Zhang, Xumei Feng, Xueyan Zhang, Tao Deng, Guanli Zhan, Ting Luo, Kui Zhang, Shuang Ding, Haibin Liu, Zhenhua Zheng, Yanyi Wang, Fang Huang, Wuxiang Guan

**Affiliations:** 1 Center for Emerging Infectious Diseases, Wuhan Institute of Virology, Chinese Academy of Sciences, Wuhan, Hubei 430071, China; 2 Hubei JiangXia Laboratory, Wuhan, Hubei 430200, China; 3 Department of Dermatology, Hangzhou Third People’s Hospital, Hangzhou, Zhejiang 310009, China; 4 Department of PathogenBiology, School of Basic Medicine, Tongji Medical College, Huazhong University of Science and Technology, Wuhan, Hubei 430030, China

**Keywords:** Immunology, Molecular biology, Virology

## Abstract

Methyltransferase-like 3 (METTL3), the core methyltransferase for *N*^6^-methyladenosine (m6A), plays a crucial role in innate immunity by introducing m6A modifications on viral or host RNAs. Despite its well-established catalytic function in m6A deposition, the broader role of METTL3 in immune regulation remains unclear. Here, we uncovered that EV71 infection enhanced METTL3 expression in interferon (IFN)-deficient Vero and IFN-proficient rhabdomyosarcoma (RD) cells by modulating transcription and post-translational modification, respectively. METTL3 was shown to regulate antiviral immune responses in both m6A-dependent and -independent manners. METTL3’s catalytic motif impaired viral RNA recognition by retinoic-acid-inducible gene I (RIG-I) via m6A modification, whereas its non-catalytic motif recruited and stabilized DEAD-box helicase 3X (DDX3X) by preventing DDX3X ubiquitination, which all mediate immune inhibition. This study reveals an m6A-independent pathway through which METTL3 regulates immune responses, highlighting its potential as a target for antiviral therapy.

## Highlights

• METTL3 exhibits distinct expression patterns in IFN-proficient and IFN-deficient cells
• METTL3 suppresses immune responses in both m6A-dependent and -independent manners
• METTL3 recruits DDX3X via its non-catalytic motif to inhibit immune response
• METTL3 enhances DDX3X expression by inhibiting its ubiquitination

## Graphical abstract

**Figure:**
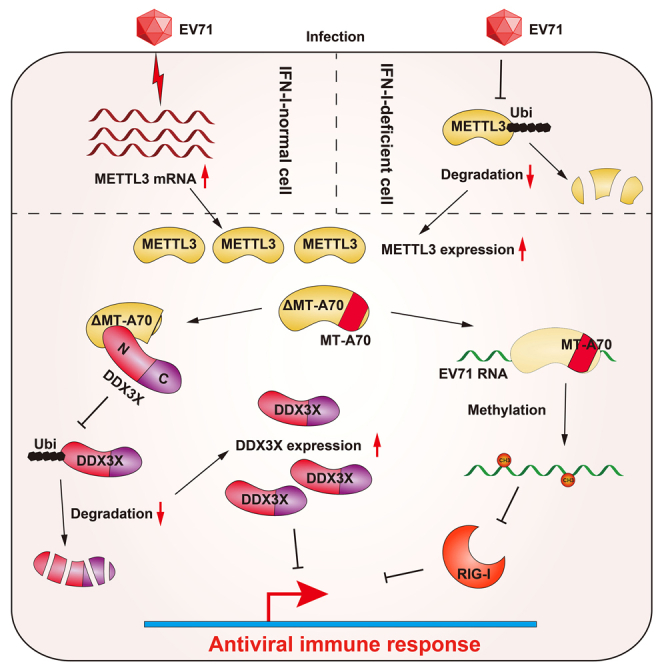

## Introduction

Chemical modification plays a critical role in the structure and function of RNA.^1^ To date, more than 180 modifications have been identified,^2^ among which, *N*^6^-methyladenosine (m6A) is the most widely prevalent modification in mRNA and long noncoding RNA (lncRNA).^3^^,^^4^^,^^5^ The m6A modification affects various RNA biological processes,^1^ including RNA stability,^6^^,^^7^^,^^8^ translation,^9^^,^^10^ splicing,^8^ nuclear export,^11^ and structure.^12^ The formation of m6A is mediated by specific methyltransferase complexes, including the three main proteins methyltransferase-like 3 (METTL3), METTL14; WT1-associated protein (WTAP); and auxiliary proteins RNA-binding motif protein 15 (RBM15), RBM15B, vir-like m6A methyltransferase associated (VIRMA), zinc finger CCCH-type containing 13 (Zc3h13), and Hakai.^13^^,^^14^^,^^15^^,^^16^^,^^17^^,^^18^^,^^19^^,^^20^^,^^21^ The dioxygenases FTO alpha-ketoglutarate-dependent dioxygenase (FTO) and AlkB homolog 5 (ALKBH5) play a crucial role in removing m6A. Moreover, the cellular functions mediated by m6A are dependent on specific m6A-binding proteins, referred to as “readers.” Among these, proteins containing the YT521-B (YTH) domain, including YTHDF1-3, YTHDC1, and YTHDC2, are the most distinctive.^8^^,^^10^^,^^22^^,^^23^^,^^24^^,^^25^^,^^26^ In addition, other host factors, including eukaryotic initiation factor 3 (eIF3), heterogeneous nuclear ribonucleoprotein (HNRNPA2B1), HNRNPC, and insulin-like growth factor 2 mRNA-binding proteins (IGF2BPs), are also characterized as the m6A readers.^9^^,^^12^^,^^27^^,^^28^ Notably, the presence of a large number of m6A readers suggests a complex cellular mechanism by which m6A modulates RNA functions.

METTL3, the core catalytic subunit within the m6A methyltransferase complex,^29^ belongs to the MT-A70 family of proteins and catalyzes methyl transfer to adenosine through its methyltransferase domain (MTD).^30^^,^^31^ The catalytic motif of METTL3 is known as DPPW.^32^ N-terminus of METTL3 contains two zinc finger domains, forming a helical structure that interacts with WTAP.^33^ The C-terminus of METTL3 contains the MTD, adopting a typical α-β-α sandwich fold and mediates the interaction between METTL3 and METTL14.^29^^,^^32^ Notably, METTL3-mediated m6A modifications play an important role in modulating the innate immune response to viral infections, including the sensing of invading RNAs and the regulation of transcripts involved in innate immune signaling.^34^ The m6A-containing RNAs inhibit Toll-like receptor (TLR) and RIG-I recognition.^35^^,^^36^ Moreover, m6A-mediated mRNA degradation regulates interferon-beta (*IFNB*) transcripts.^37^ However, it remains uncertain whether the METTL3 protein, beyond its role in methyltransferase activity, plays a direct role in regulating innate immunity.

Innate immunity, activated in the early stage of viral infections, serves as the first line of defense against pathogens. Viral nucleic acids, recognized as pathogen-associated molecular patterns, are detected by pattern recognition receptors (PRRs).^38^ The DEAD-box helicase (DDX) protein family plays an important role in the antiviral innate immune response, and some members of the family are associated with the m6A modification. DDX46 demethylates the transcripts of mitochondrial antiviral signaling protein (*Mavs*), tumor necrosis factor (TNF)-receptor-associated factor 3 (*Traf3*), and *Traf6* by recruiting ALKBH5. This recruitment ensures the nuclear retention of these RNAs, thus inhibiting the translation of the indicated proteins.^39^ DDX5 regulates m6A levels on the antiviral transcripts, enhancing RNA decay to suppress antiviral innate immunity.^40^^,^^41^ Recently, DDX3X was reported to exhibit significant, albeit ambiguous roles, in viral infection.^42^^,^^43^ Nevertheless, DDX3X is able to promote the replication of human immunodeficiency virus (HIV), hepatitis C virus (HCV), and some other viruses^44^^,^^45^^,^^46^^,^^47^^,^^48^^,^^49^ but resist dengue virus (DENV) and hepatitis B virus (HBV).^50^^,^^51^^,^^52^^,^^53^ Despite evidence suggesting DDX3X as a promising antiviral target, currently available DDX3X inhibitors, suppressing its ATPase or helicase activity, exert a substantial impact on the overall host RNA metabolism.^42^^,^^54^ Therefore, elucidating the key interactions of DDX3X with viral proteins and other host factors will pave the way for the development of antiviral drugs targeting DDX3X protein-protein interactions.

The present study aimed to comprehensively investigate two distinct pathways underlying the regulation of EV71-induced immune responses by METTL3. Specifically, the regulatory effects of m6A catalysis, facilitated by the MT-A70 domain of METTL3 and the interactions between METTL3 and DDX3X via its non-catalytic motif, on innate immunity were revealed. This study proposes a mechanism by which METTL3 regulates the immune responses and characterizes the function of METTL3 beyond its enzyme activities. These findings present potential therapeutic avenues for blocking RNA viral infections.

## Results

### METTL3 expression pattern differs between normal and IFN-deficient cells

**Figure 1:**
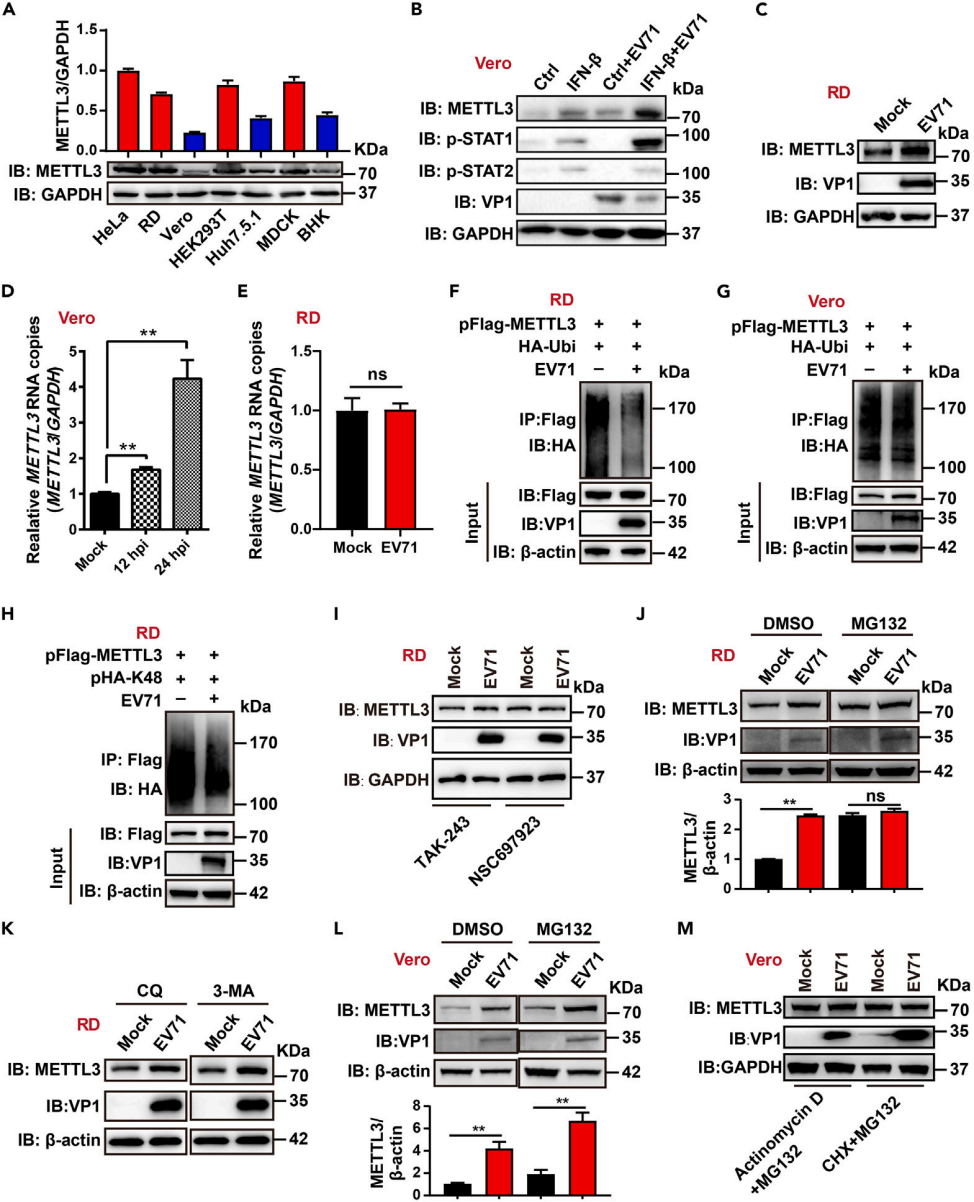
EV71 enhances METTL3 expression at the transcriptional and post-transcriptional levels (A) Expression levels of METTL3 in different cell lines. The various cells were lysed and subjected to western blotting using antibodies against METTL3. GAPDH was used as a loading control. Relative intensity of METTL3 versus GAPDH was quantified using the ImageJ program and shown above. Data are means ± SDs (n = 3). (B) Vero cells were treated with IFN-β (200 ng/mL) or infected with EV71. Western blot was performed using the indicated antibodies. GAPDH served as a loading control. (C) The expression of METTL3 in mock- or EV71-infected RD cells was assessed by western blot using anti-METTL3 antibodies. GAPDH served as a loading control. (D and E) Relative RNA copies of METTL3 in mock- or EV71-infected Vero (D) or RD (E) cells were quantified by RT-qPCR, with *GAPDH* as a control. Data are means ± SEMs (n = 3). ∗∗*p* ≤ 0.01, ns: not significant, unpaired Student’s t test. (F–H) Ubiquitination assay. RD or Vero cells were transfected with pFlag-METTL3 and pHA-Ub (F and G) or pHA-K48 (H) and infected with EV71 (MOI = 1). IP and immunoblot analysis were performed using the indicated antibodies. (I) RD cells were infected with EV71 for 10 h and then treated with TAK-243 (500 nM) or NSC697923 (20 μM) for 6 h. The expression of METTL3 was detected by western blot. (J and L) METTL3 expression in mock- or EV71-infected RD (J) or Vero (L) cells treated with DMSO or 20 μM MG132 was detected by western blot using anti-METTL3 antibodies, and β-actin was used as a loading control. Relative intensity of METTL3 versus β-actin was quantified using the ImageJ program. Data are means ± SDs (n = 3). ∗∗*p* ≤ 0.01, ns: not significant, unpaired Student’s t test. (K) Mock- or EV71-infected-RD cells were treated with CQ (10 μM) or 3-MA (5 mM) for 6 h. METTL3 expression was detected by western blot. (M) Mock- or EV71-infected Vero cells were treated with actinomycin D (4 μg/mL) plus MG132 (20 μM) or CHX (100 μg/mL) plus MG132 (20 μM) for 6 h, and METTL3 expression were detected by immunoblot assay.

METTL3 regulates innate immune responses by affecting RNA m6A modifications.^55^ To investigate the association between METTL3 expression and antiviral innate immunity, we measured the expression levels of METTL3 in interferon (IFN)-normal cells (HeLa, RD, HEK293T, and MDCK) and IFN-deficient cells (Vero, BHK, and Huh7.5.1) (Figure 1A). METTL3 protein levels were considerably lower in IFN-deficient cells than in normal cells (Figure 1A). Furthermore, the transcription, translation, and degradation of METTL3 in these cells were detected. RT-qPCR assays showed that METTL3 RNA levels were significantly lower in Huh7.5.1, Vero, and BHK compared to IFN-normal cells (Figure S1A). Ribosomal loading experiments revealed no notable differences in METTL3 translation levels across these cell types (Figure S1B). Additionally, METTL3 expression in IFN-deficient cells did not exhibit a more obvious increase than that in IFN-normal cells after MG132 treatment (Figure S1C). Overall, the reduced expression of METTL3 in IFN-deficient cells is primarily attributed to lower transcription levels.

Next, the effect of IFN on METTL3 expression was examined. Upon the addition of IFN-β to Vero cells, the expression of METTL3 was upregulated, accompanied by increased phosphorylation of STAT1 and STAT2 (Figure 1B). Moreover, EV71 infection synergistically increased METTL3 expression in the presence of IFN-β (Figure 1B). The increased METTL3 expression was also observed in EV71-infected rhabdomyosarcoma (RD) cells (Figure 1C). These data indicated that METTL3 expression pattern was closely related to IFN-I and EV71 infection.

To investigate the mechanism underlying EV71-induced upregulation of METTL3 expression, we quantified the transcription levels of *METTL3* in EV71-infected Vero and RD cells. In Vero cells, *METTL3* RNA abundance was increased at 12 h and 24 h after EV71 infection (Figures 1D and S1D), whereas no significant differences were observed in EV71-infected RD cells (Figures 1E and S1E), suggesting that distinct mechanisms might be utilized by EV71 to modulate METTL3 expression in these two types of cells. METTL3 expression was reported to be affected by ubiquitination.^56^ We subsequently compared the levels of METTL3 ubiquitination in different cells with or without EV71 infection. Notably, METTL3 ubiquitination was suppressed in EV71-infected RD and HEK293T cells (Figures 1F and S1F) but not in Vero cells (Figure 1G). Our previous study demonstrated that SARS-CoV-2 influences both K48- and K63-linked ubiquitination of METTL3.^57^ This led us to speculate that EV71 might similarly impact these types of METTL3 ubiquitination. Indeed, EV71 infection obviously inhibited the K48 ubiquitination of METTL3 in RD and HEK293T cells (Figures 1H and S1G), and a weak inhibition of K48 ubiquitination was also observed in Vero cells (Figure S1G). Conversely, K63-linked ubiquitination of METTL3 in HEK293T cells slightly increased after EV71 infection (Figure S1H). To further validate these results, RD cells were pre-treated with the ubiquitination inhibitors TAK-243 or NSC697923 prior to EV71 infection. TAK-243 or NSC697923 treatment effectively blocked METTL3 ubiquitination (Figure S1I) and maintained METTL3 expression levels regardless of EV71 infection (Figure 1I).

K48-linked ubiquitination of target proteins typically results in proteasome-mediated degradation.^58^ We used MG132, a proteasome inhibitor, to block the proteasome pathway and observed that EV71 no longer increased METTL3 expression in RD cells (Figure 1J). Moreover, chloroquine (CQ) and 3-methyladenine (3-MA) were separately employed to inhibit the autophagy and lysosomal pathways, and EV71 still upregulated METTL3 expression (Figure 1K). To exclude the possibility that EV71 directly affects METTL3 translation, the ribosome inhibitor cycloheximide (CHX) was added to RD cells, and it was found that EV71 infection enhances METTL3 expression (Figure S1J). These results indicate that EV71 promotes METTL3 expression in RD cells by inhibiting the proteasome degradation pathway. In Vero cells, a synergistic effect of METTL3 expression was observed upon MG132 treatment and EV71 infection (Figure 1L). However, when transcription and translation were inhibited with actinomycin D or CHX, EV71 infection no longer enhanced METTL3 expression in Vero cells (Figure 1M). Taken together, these results revealed that EV71 infection enhanced METTL3 expression in Vero and RD cells mainly via transcriptional and post-translational mechanisms, respectively.

### METTL3 inhibits EV71-induced IFN-I response

**Figure 2:**
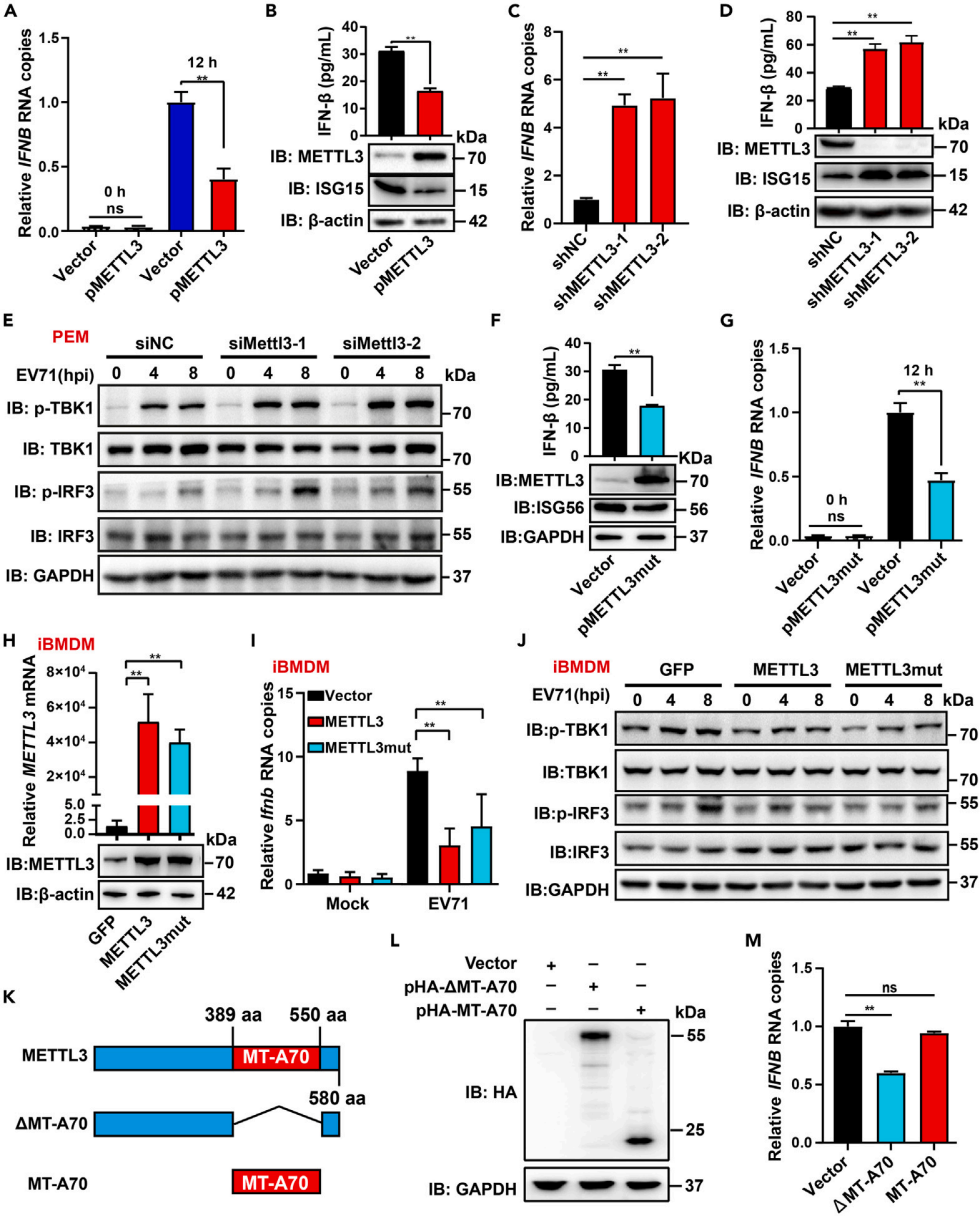
METTL3 inhibits EV71-induced antiviral immune responses in an m6A-dependent and -independent manner (A, C, and G) pMETTL3- (A), shMETTL3- (C), or pMETTL3mut (DPPW-APPA, G)-treated RD cells were infected with EV71 at indicated times and the RNA levels of *IFNB* were quantified by RT-qPCR. Data are means ± SEMs (*n* = 3). ∗∗*p* ≤ 0.01, ns, not significant, unpaired Student’s t tests. (B, D, and F) RD cells were treated with pMETTL3 (B), shMETTL3 (D), or pMETTL3mut (F) and then infected with EV71 for 12 h. The expression of METTL3, ISG15, and ISG56 were detected by western blot, and the concentrations of IFN-β in cell culture supernatants were examined by ELISA. Data are means ± SDs (*n* = 3). ∗∗*p* ≤ 0.01, unpaired Student’s t tests. (E) Specific siRNAs (40 nM) were used to knock down *Mettl3* in PEMs, followed by EV71 infection. The phosphorylation levels of TBK1 and IRF3 were measured by western blotting. (H) iBMDMs were stably transfected with METTL3 or METTL3mut using retrovirus to detect METTL3 expression by RT-qPCR and western blot. RT-qPCR data are means ± SEMs (*n* = 3). ∗∗*p* ≤ 0.01, unpaired Student’s t tests. (I) iBMDMs-METTL3 and iBMDMs-METTL3mut were infected with EV71; the RNA levels of *Ifnb* were quantified by RT-qPCR. Data are means ± SEMs (*n* = 3). ∗∗*p* ≤ 0.01, two-way ANOVA with Holm-Sidak’s multiple comparisons test. (J) iBMDMs-METTL3/METTL3mut were infected with EV71 for different hours to detect the phosphorylation levels of TBK1 and IRF3. (K) Schematic diagram of truncated METTL3. (L) The expression of MT-A70 and ΔMT-A70 was assessed by western blotting using anti-HA antibodies. (M) Vector-, pΔMT-A70-, and pMT-A70-transfected-HEK293T cells were infected with EV71 to detect the RNA levels of *IFNB* by RT-qPCR. Data are means ± SEMs (*n* = 3). ∗∗*p* ≤ 0.01, ns: not significant, unpaired Student’s t tests.

EV71 infection activates the host’s immune responses and induces IFN production, which in turn blocks viral replication.^59^ To figure out whether METTL3 could regulate EV71-induced immune responses, RD cells with overexpression or depletion of METTL3 were infected with EV71 to detect the levels of inflammatory factors. Exogenous METTL3 inhibited mRNA levels of *IFNB*, *IFNA*, and *ISG15* (Figures 2A and S2A) and suppressed protein levels of IFN-α, IFN-β, and ISG15 (Figures 2B and S2B)*.* Consistently, knockdown of METTL3 resulted in increased mRNA and protein levels of IFNB*,* IFNA, and ISG15 (Figures 2C, 2D, S2C, and S2D). Besides, METTL3 also inhibited the transcription of *IFNB*, *IFNA*, and *ISG15* in EV71-infected HEK293T and HeLa cells (Figures S2E and S2F). To further confirm these, specific small interfering RNAs (siRNAs) were used to knock down *Mettl3* in peritoneal exudate macrophages (PEMs) (Figure S2G) followed by EV71 infection. Silence of *Mettl3* obviously upregulated the transcription of *Ifnb*, *Isg15*, and *Isg56* (Figure S2H), increased the secretion of IFN-β (Figure S2I), and enhanced the phosphorylation levels of TBK1 and IRF3 (Figure 2E). Moreover, the METTL3 inhibitor STM2457 also improved the mRNA levels of *Ifnb*, *Isg15*, and *Isg56* (Figure S2J).

Furthermore, the role of METTL3 in Sendai virus (SeV)-infected HEK293T cells was detected. Luciferase assay showed that METTL3 overexpression attenuated the activation of *IFNB*, ISRE, and nuclear factor κB (NF-κB) (Figure S3A). In contrast, *METTL3* knockdown promoted SeV-induced mRNA levels of *IFNB*, *ISG56*, *RIG-I*, and *IP10* (Figures S3B–S3E). Collectively, METTL3 was able to negatively regulate EV71- and SeV-induced IFN-I responses.

METTL3-catalyzed m6A modifications have been demonstrated to inhibit immune responses.^60^ To investigate whether its methyltransferase activity dominated the METTL3-mediated inhibition of immune responses, mutant METTL3 (pMETTL3mut, DPPW-APPA) with aberrant catalytic motif was constructed and introduced into RD cells (Figure 2F). Nevertheless, METTL3mut overexpression also resulted in decreased mRNA and protein levels of IFNA, IFNB, and ISG56 (Figures 2F, 2G, S4A, and S4B). To further ascertain this phenotype, we constructed immortalized bone-marrow-derived macrophages (iBMDMs) that stably expressed human METTL3 or METTL3mut (Figure 2H). Both METTL3 and METTL3mut inhibit the expression of IFN-β (Figures 2I and S4C). Similar to METTL3 overexpression, METTL3mut also reduced the phosphorylation levels of TBK1 and IRF3 after EV71 infection (Figure 2J). Together, these results indicated that the methyl transfer function of METTL3 was not indispensable for its inhibitory effects on immune responses.

The MT-A70 domain determines the methyltransferase activities of METTL3 and contains the catalytic motif DPPW. To further verify the aforementioned implication, two truncated METTL3 mutants, deleting the MT-A70 domain (ΔMT-A70) or containing only the MT-A70 motif (NCBI Reference Sequence: NP_062826.2), were constructed (Figure 2K) and overexpressed in RD cells (Figure 2L). Notably, only the ΔMT-A70 mutant led to an inhibited transcription of *IFNA*, *IFNB*, and *ISG56* (Figures 2M and S4D) compared to the empty vector and the MT-A70 mutant. Taken together, METTL3 was capable to suppress IFN-I responses independently of its methyl-transfer function.

### EV71 m6A leads to impaired RIG-I recognition and decreased immune responses

**Figure 3:**
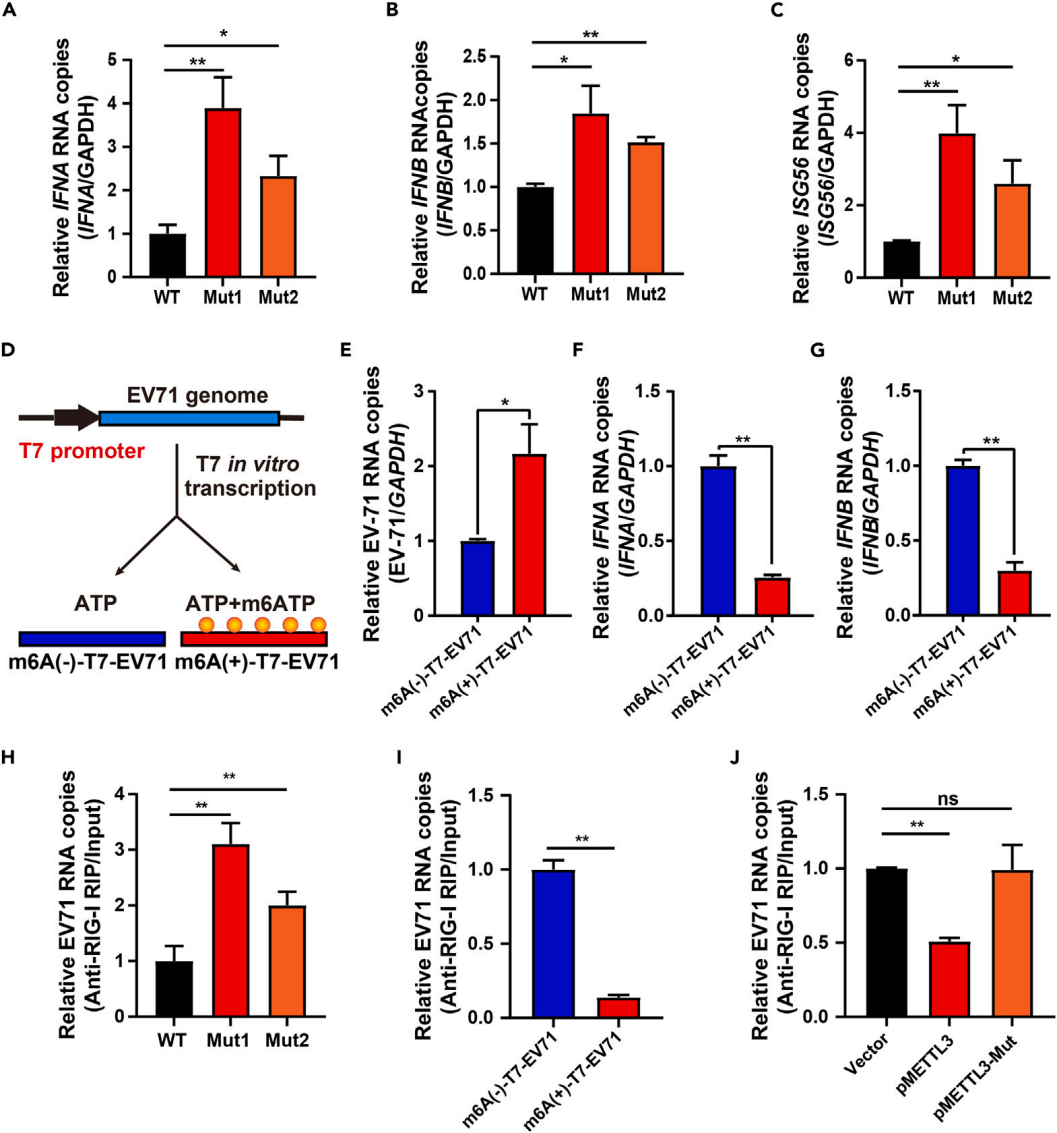
METTL3-catalyzed EV71 m6A suppresses immune response by inhibiting RIG-I recognition (A–C) RD cells were infected with WT or m6A-mut EV71 (MOI = 1), and the RNA levels of *IFNA* (A), *IFNB* (B), and *ISG56* (C) were quantified by RT-qPCR. Data are means ± SEMs (*n* = 3). ∗*p* ≤ 0.05, ∗∗*p* ≤ 0.01, unpaired Student’s t tests. (D) Schematic representation of the m6A(±) T7-EV71 RNAs. Yellow solid circles indicate m6A modification. (E–G) Total RNA of m6A(±) T7-EV71-transfected RD cells were extracted at 12 h post-transfection. The RNA levels of EV71 (E), *IFNA* (F), and *IFNB* (G) were quantified by RT-qPCR. Data are means ± SEMs (*n* = 3). ∗*p* ≤ 0.05, ∗∗*p* ≤ 0.01, unpaired Student’s t tests. (H–J) m6A modification blocked RIG-I binding to EV71 RNA. RD cells were infected with WT or m6A-mut EV71 (H), transfected with m6A(±) T7-EV71 (I), or transfected with pMETTL3/pMETTL3mut prior to EV71 infection (J). Cells were formaldehyde crosslinked and immunoprecipitated with anti-RIG-I antibodies, followed by qRT-PCR. Data are means ± SEMs (*n* = 3). ∗∗*p* ≤ 0.01, ns, not significant, unpaired Student’s t tests.

METTL3 was previously demonstrated to catalyze m6A modifications on EV71 RNA, which further promoted viral replication.^61^^,^^62^^,^^63^ m6A-modified RNAs were found to impair the immune responses compared to unmodified RNAs^35^^,^^36^; however, whether m6A affects EV71-induced innate immunity remains unclear. To address this, RD cells were infected with wild-type (WT) or two different m6A-mutant EV71 (m6A-mut1 and m6A-mut2).^61^ Compared to WT viruses, infection with either m6A-mutant EV71 enhanced the mRNA levels of *IFNA*, *IFNB*, and *ISG56* (Figures 3A–3C). Furthermore, *in vitro* T7 transcribed EV71 genomes with ATP (m6A[−]) or m6ATP (m6A[+]) as substrates were separately introduced into RD cells (Figure 3D). The incorporation of m6A modifications onto the genome RNAs of EV71 not only enhanced the subsequent viral replication efficiency (Figure 3E) but also decreased the mRNA levels of *IFNA*, *IFNB* (Figures 3F and 3G), *ISG15*, *ISG54*, *IL1B*, *IL6*, *IL8*, and *IL18* (Figures S5A–S5F). Thus, these results implied that m6A modifications attenuated the immune responses activated by EV71 infection.

RIG-I is a pivotal PRR responsible for recognizing pathogenic RNAs in infected cells and inducing IFN-I responses.^64^^,^^65^^,^^66^^,^^67^^,^^68^ However, m6A-modified RNAs inhibit the binding of RIG-I.^35^ To investigate whether METTL3-catalyzed m6A modifications on EV71 RNAs suppressed the immune response by impairing RIG-I recognition, we infected RD cells with WT or two m6A mutant EV71. RNA immunoprecipitation (RIP) analysis using RIG-I antibodies revealed that mutant EV71 RNAs exhibited stronger affinity to RIG-I than that of WT (Figure 3H). Moreover, *in vitro* transcribed m6A(+)-T7-EV71 genomes showed a lower RIG-I binding capacity than that of their unmodified counterparts (Figure 3I). Exogenous METTL3 led to decreased binding of RIG-I to viral RNA in EV71-infected RD cells, whereas METTL3mut did not exhibit this effect (Figures 3J and S6A). A similar phenomenon was also observed in HEK293T cells (Figures S6B–S6D). Overall, METTL3-mediated m6A modifications on EV71 RNAs diminished the recognition of viral RNAs by RIG-I, thereby attenuating EV71-induced IFN-I responses.

### ΔMT-A70 region of METTL3 interacts with DDX3X

**Figure 4:**
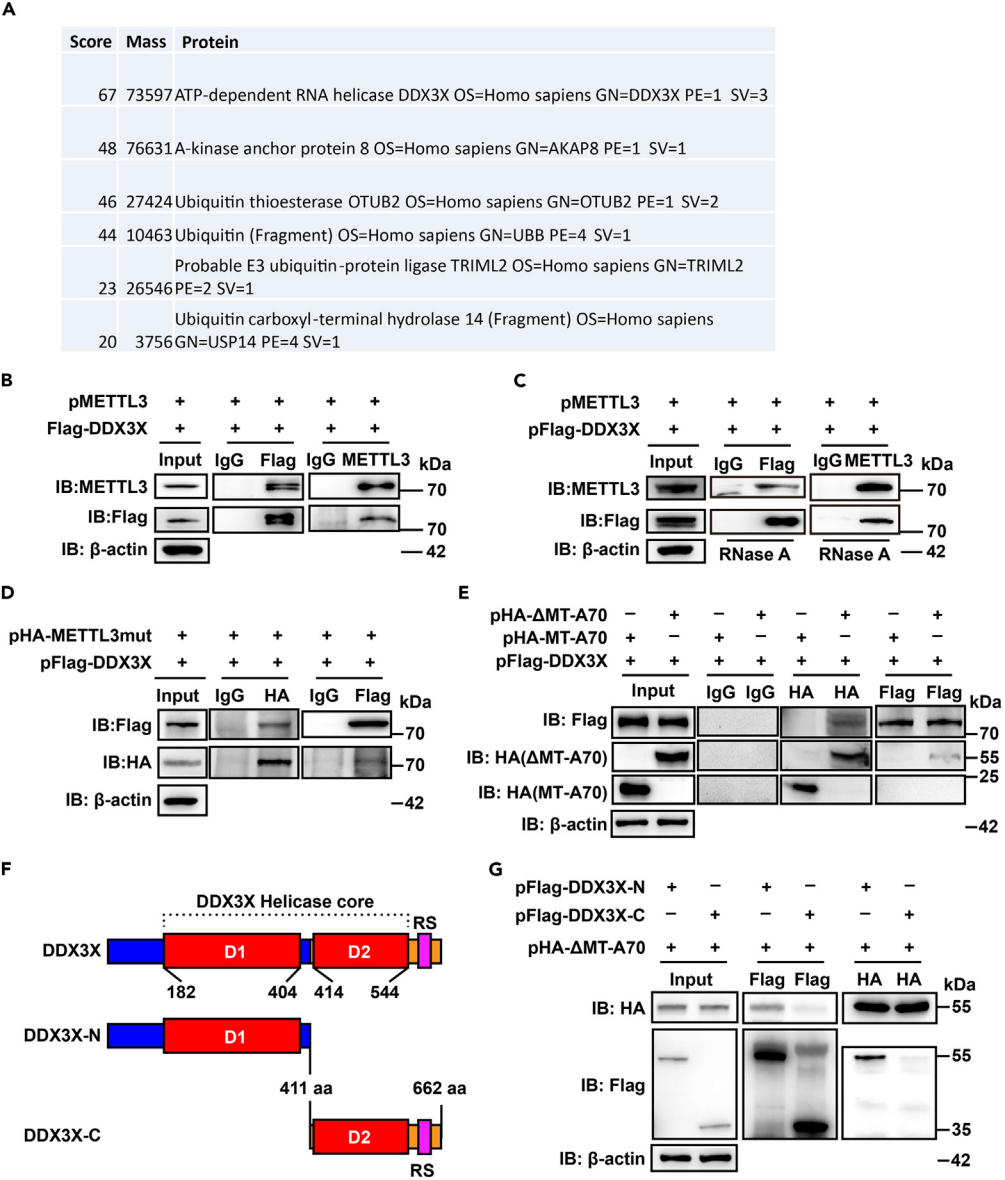
METTL3 interacts with DDX3X (A) Immune-related proteins that interact with METTL3. pFlag-METTL3-transfected RD cells were infected with EV71 and subjected to IP using anti-Flag antibody and analyzed by MS analysis. (B) HEK293T cells were transfected with pFlag-DDX3X and pMETTL3 followed by co-IP with immunoglobulin G (IgG) or anti-Flag (left) or anti-METTL3 (right) antibodies. The immune blots were probed using western blotting with the anti-Flag or anti-METTL3 antibodies. (C) The same experimental operation was performed as in (B), except that the cell extracts were digested with RNase A at 37°C for 15 min before co-IP. (D and E) pFlag-DDX3X co-transfected with pMETTL3mut (catalytic center mutant) (D) or pΔMT-A70 or pMT-A70 into HEK293T cells and followed by the same experimental operation as (B). (F) Schematic diagram of truncated DDX3X. D1, RecA-like domains 1; D2, RecA-like domains 2; RS, arginine-serine-rich domain. (G) HEK293T cells were transfected with pHAΔMT-A70 and pFlag-DDX3X-N or pFlag-DDX3X-C, followed by the same experimental operation as (B).

To explore the potential mechanisms underlying the inhibitory effects of METTL3 on innate immunity independent of its m6A catalytic activities, METTL3 immunoprecipitates from EV71-infected RD cells were obtained and analyzed by mass spectrometry. The identified METTL3-associated proteins included the immune-related proteins DDX3X (ranking as the 1st candidate), AKAP8, and some ubiquitin-related proteins (Figure 4A). The interactions between DDX3X and METTL3 were further validated by co-immunoprecipitation (co-IP) assays (Figure 4B). Given that both DDX3X and METTL3 target RNAs for binding, to elucidate whether such interactions between these two proteins were RNA dependent, RNase A was introduced into the above co-IP assays. After digestion with RNase A, METTL3 still interacted with DDX3X at a similar level (Figure 4C), indicating that such interactions were independently of RNA.

As the catalytic motif of METTL3 has been revealed to be not dispensable for the inhibitory effects of immune responses (Figure 2M), DDX3X targeted by METTL3 could potentially mediate such inhibition independent of m6A catalytic activities. Therefore, we detected and validated the interactions between METTL3mut and DDX3X via co-IP assays (Figure 4D). In addition, DDX3X interacted with the ΔMT-A70 region of METTL3 but not MT-A70 (Figure 4E). Furthermore, truncated DDX3X mutants were constructed to characterize the exact motif interacting with METTL3. The helicase core of DDX3X contains two domains, RecA-like domain 1 (D1) and RecA-like domain 2 (D2),^69^^,^^70^ which were included in DDX3X-N and DDX3X-C, respectively (Figure 4F). As presented in Figure 4G, the N terminus of DDX3X determined its interaction with METTL3. Taken together, METTL3 targeted DDX3X for binding, which was not dependent on the RNA and m6A catalytic activities of METTL3.

### DDX3X is essential for the m6A-independent function of METTL3 to inhibit IFN-I

**Figure 5:**
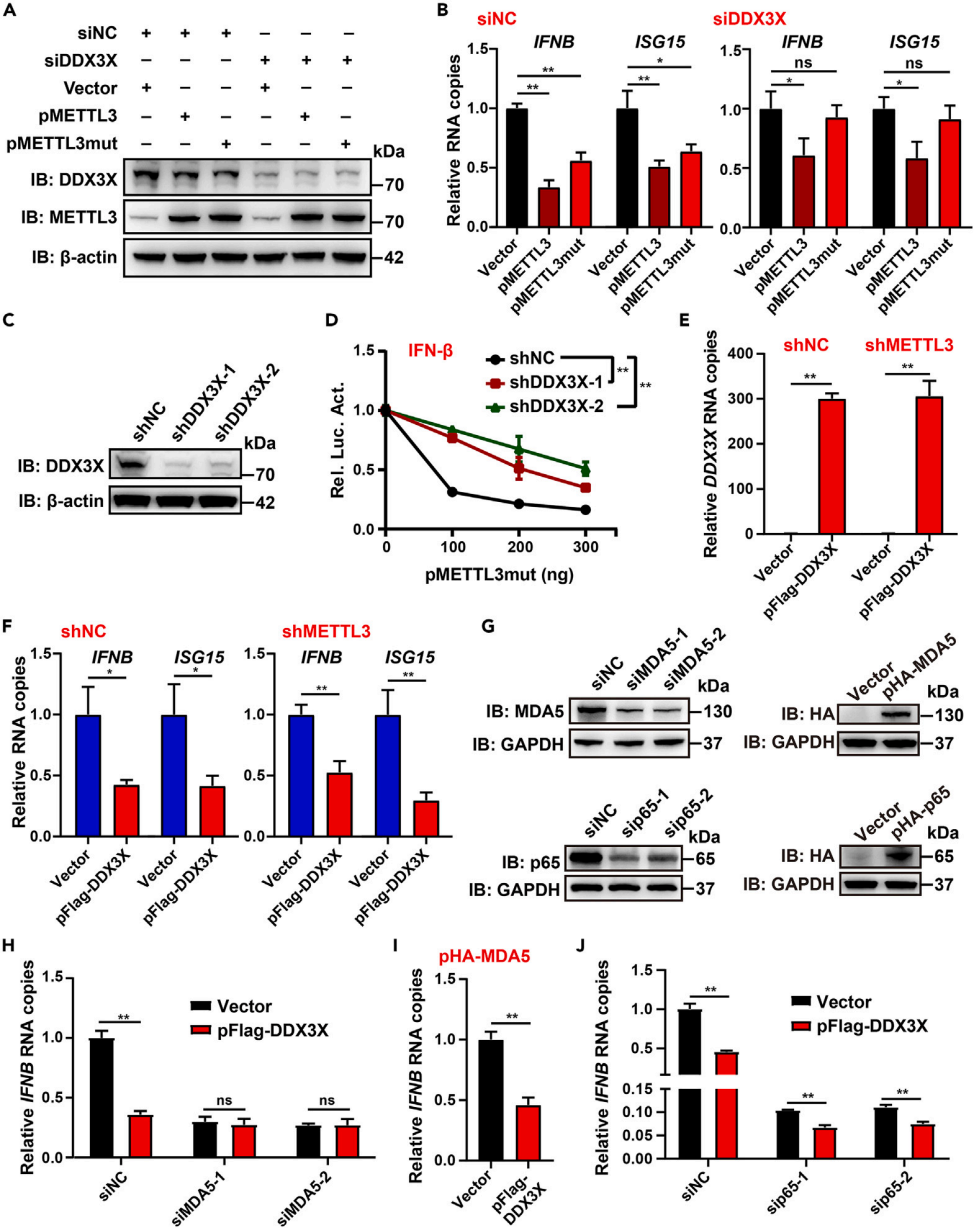
DDX3X regulates immune responses (A) Vector, pMETTL3, or pMETTLmut were co-transfected with siNC or siDDX3X (the mixture of siDDX3X-1 and siDDX3X-2) into RD cells, followed by EV71 infection. The expression of DDX3X and METTL3 was assessed using western blotting with the indicated antibodies. (B) The transcripts of *IFNB* and *ISG15* in the RNAs collected from the cells in (A) were quantified by RT-qPCR. Data are means ± SEMs (*n* = 3). ∗*p* ≤ 0.05, ∗∗*p* ≤ 0.01, ns: not significant, unpaired Student’s t tests. (C) RD cells were treated with shNC or shDDX3X. The knockdown efficiency was confirmed by western blotting. (D) DDX3X-knockdown RD cells were co-transfected with IFN-β reporter plasmids and increasing concentration of pMETTL3mut. Luciferase assays were performed after the EV71 infection. Data are means ± SEMs (*n* = 3). ∗∗*p* ≤ 0.01, two-way ANOVA. (E and F) Vector or pFlag-DDX3X was transfected into shNC- or shMETTL3 (the mixture of shMETTL3-1 and shMETTL3-2)-treated RD cells, followed by EV71 infection. The transcripts of *DDX3X*, *IFNB*, and *ISG15* were detected by RT-qPCR. Data are means ± SEMs (*n* = 3). ∗*p* ≤ 0.05, ∗∗*p* ≤ 0.01, unpaired Student’s t tests. (G) siRNAs and expression plasmids for MDA5 and p65 were transfected to RD cells, respectively. The expression of MDA5 and p65 were detected by western blotting. (H–J) Effects of DDX3X on *IFNB* transcription in cells treated with siNC, siMDA5 (H), sip65 (J), or pHA-MDA5 (I) were detected using RT-qPCR. Data are means ± SEMs (*n* = 3). ∗∗*p* ≤ 0.01, ns: not significant, unpaired Student’s t tests.

DDX3X was reported to regulate innate immune responses^71^ and was found to bind to the ΔMT-A70 region of METTL3 (Figure 4E), a non-catalytic motif that was essential for the inhibitory effects of IFN-I-related immune responses (Figure 2M). Therefore, we posited that DDX3X could be the mediator among the pathways of METTL3-inhibited immune responses. To test this hypothesis, METTL3 or METTL3mut was overexpressed in RD cells with or without *DDX3X* knockdown, followed by EV71 infection (Figure 5A). Transfection with either METTL3 or METTL3mut inhibited EV71-induced transcription levels of *IFNB* and *ISG15* in negative control (NC) siRNA-transfected cells, with the former exhibiting stronger effects (Figure 5B). In contrast, in *DDX3X* siRNA-treated cells, the inhibitory effect of METTL3 on *IFNB* and *ISG15* mRNA levels was moderately attenuated, whereas the inhibitory effects of METTL3mut were completely blocked (Figure 5B). Furthermore, the reduction of IFN-β luciferase activities induced by exogenous METTL3mut was obviously restored in shDDX3X-transfected RD cells compared with shNC-transfected cells (Figures 5C and 5D). To further characterize the exact role of DDX3X in the METTL3-mediated inhibition of immune responses, exogenous DDX3X was overexpressed in shNC- or shMETTL3-transfected RD cells, followed by EV71 infection. The mRNA levels of *DDX3X* were not affected by METTL3 depletion (Figure 5E). However, exogenous DDX3X decreased *IFNB* and *ISG15* mRNAs at similar levels in both shNC- and shMETTL3-transfected RD cells (Figure 5F). Thus, DDX3X functioned downstream of the IFN-I response cascade mediated by the non-m6A catalytic activity of METTL3.

DDX3X interacts with MAVS, IKKε, TBK1, and IRF3, thereby enhancing IFN-I responses.^72^^,^^73^^,^^74^^,^^75^ However, DDX3X has also been shown to suppress immune responses by interacting with the NF-κB subunit p65 or by enhancing the cytoplasmic accumulation of endogenous dsRNA to boost the melanoma-differentiation-associated gene 5 (MDA5)-mediated dsRNA-sensing pathway.^76^^,^^77^ Since both NF-κB and MDA5 are involved in the immune response triggered by EV71,^59^^,^^78^ we hypothesized that p65 and MDA5 might play roles in the regulatory effect of DDX3X on EV71-induced immune response. To test this, we used siRNA and expression plasmids to knock down or overexpress p65 or MDA5 in RD cells (Figure 5G). Our findings showed that the inhibitory effect of DDX3X on *IFNB* transcription was abolished in MDA5 knockdown cells after EV71 infection (Figure 5H). In contrast, DDX3X continued to inhibit the IFN response in MDA5 overexpressing cells (Figures 5I and S7A). Furthermore, DDX3X inhibited EV71-induced *IFNB* expression regardless of p65 overexpression or knockdown (Figures 5J and S7B). These results indicate that DDX3X may primarily suppress the EV71-induced immune response by inhibiting MDA5 activation.

### METTL3 enhances the stability of DDX3X by inhibiting its ubiquitination

**Figure 6:**
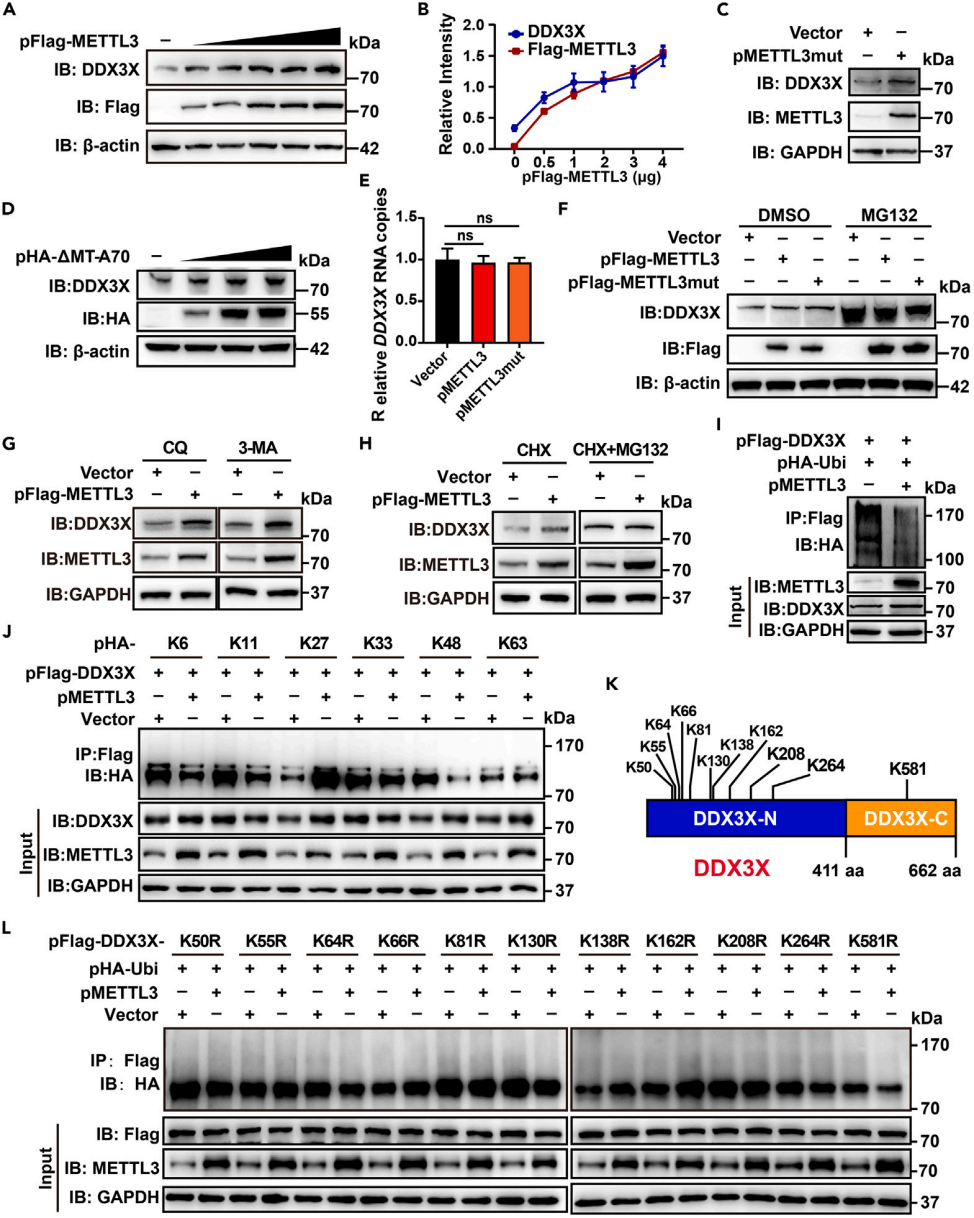
METTL3 promotes DDX3X expression by affecting its ubiquitination (A) HEK293T cells were transfected with pFlag-METTL3 (0, 0.5, 0.75, 1, 2, and 4 μg, respectively) in 6-well plates, and missing plasmids in each well were replenished using Vector. The expression of DDX3X and Flag-METTL3 was detected by western blot using antibodies against Flag and METTL3. GAPDH was used as a loading control. (B) Relative intensity of DDX3X or METTL3 versus GAPDH was quantified using the ImageJ program. Data are means ± SDs (n = 3). (C) HEK293T cells were transfected with vector or pMETTL3mut. The expression of DDX3X and METTL3mut was detected by western blot using antibodies against DDX3X and METTL3. GAPDH was used as a loading control. (D) HEK293T cells were transfected with pHA-ΔMT-A70 (0, 0.5, 1, 2 μg, respectively) in 12-well plates. The expression of DDX3X and HA-ΔMT-A70 was detected by western blot using the indicated antibodies. GAPDH was used as a loading control. (E) Total RNA was extracted from HEK293T cells transfected with the vector, pMETTL3, or pMETTL3mut. Quantification of the RNA expression of DDX3X via RT-qPCR, with *GAPDH* used as a control. Unpaired Student’s t test was performed, and data are presented as the means ± SEMs (n = 3). ns: not significant. (F) Vector, pMETTL3, or pMETTL3mut were transfected into HEK293T cells and then treated with DMSO or 20 μM MG132. The expression of METTL3 and DDX3X were detected by western blotting. (G and H) Vector- or pFlag-METTL3-transfected-HEK293T cells were treated with 10 μM CQ, 5 mM 3-MA (G), 100 μg/mL CHX, or 20 μM MG132 (H) for 6 h. DDX3X expression was detected by immunoblot. (I and J) Ubiquitination assay. HEK293T cells were transfected with pFlag-DDX3X, vector, pMETTL3, pHA-Ub (I), pHA-K6, -K11, -K27, -K33, -K48, or -K63 (J). IP and immunoblot analysis were performed using the indicated antibodies. (K) Diagram of the ubiquitination sites of DDX3X. (L) pHA-Ubi and vector or pMETTL3 were co-transfected with pDDX3X-K50R, -K55R, -K64R, -K66R, -K81R, -K130R, -K138R, -K162R, -K208R, -K264R, or -K581R to detect the ubiquitination of DDX3X.

The transcription levels of exogenous DDX3X were not affected by METTL3 (Figure 5E), indicating the presence of other post-transcriptional regulatory mechanisms of METTL3 on DDX3X. After increasingly overexpressing METTL3 in RD and HEK293T cells, the expression levels of endogenous DDX3X were increased in a dose-dependent manner (Figures 6A, 6B, and S8A). Similarly, the exogenous METTL3mut also resulted in upregulated DDX3X expression (Figure 6C). Since the interaction between METTL3 and DDX3X were determined by the non-catalytic domains, we measured the expression levels of DDX3X in HEK293T cells overexpressing ΔMT-A70 or MT-A70 of METTL3. DDX3X expression was enhanced by transfection with the ΔMT-A70 region of METTL3 in a dose-dependent manner (Figure 6D) but not affected by transfection with the MT-A70 (Figure S8B). These data suggest that METTL3 not only interacted with DDX3X but also promoted DDX3X expression.

To elucidate the mechanism by which METTL3 promoted DDX3X expression, mRNA levels of endogenous DDX3X were examined in the presence of exogenous METTL3 or METTL3mut (Figure S8C). Neither METTL3 nor METTL3mut affected the transcription levels of *DDX3X* (Figure 6E). DDX3X is reportedly ubiquitinated, leading to its degradation.^79^ Thus, METTL3- or METTL3mut-transfected HEK293T cells were treated with MG132, which generally enhanced DDX3X protein levels; however, neither METTL3 nor METTL3mut further promoted DDX3X expression compared to vector control (Figure 6F). Additionally, DDX3X expression remained elevated following METTL3 overexpression regardless of CQ and 3-MA treatment (Figure 6G). Furthermore, CHX treatment increased DDX3X protein levels in METTL3-transfected HEK293T cells, but this enhancement was no longer observed after addition of MG132 (Figure 6H). Together, these results indicated that METTL3 mainly inhibited the proteasome-pathway-mediated degradation, thereby enhancing DDX3X expression.

To investigate the effect of METTL3 on DDX3X ubiquitination, HEK293T cells were co-transfected with DDX3X and ubiquitin, with/without METTL3 or METTL3mut. Both exogenous METTL3 and METTL3mut reduced the levels of ubiquitinated DDX3X (Figures 6I and S8D). DDX3X undergoes various types of ubiquitination, including K6, K11, K27, K33, K48, and K63 linkages.^69^^,^^80^ To determine which specific ubiquitin chains on DDX3X are influenced by METTL3, plasmids encoding ubiquitin linked to these lysine residues were used. As shown in Figure 6J, overexpression of METTL3 primarily decreased K6-, K11-, and K48-linked ubiquitin chains on DDX3X. To pinpoint the specific residues of DDX3X targeted by METTL3 for ubiquitination, we constructed DDX3X mutant clones with substitutions at various lysine residues (K50R, K55R, K64R, K66R, K81R, K130R, K138R, K162R, K208R, K264R, and K581R) based on prior literatures^69^^,^^80^ (Figure 6K). METTL3 overexpression reduced ubiquitination at DDX3X mutants K50R, K64R, K130R, K208R, K264R, and K581R (Figure 6L), indicating that METTL3 has minimal impact on these sites. Therefore, METTL3 mainly affected the ubiquitination of DDX3X at K55, K66, K81, K138, and K162. Notably, ubiquitination modification at the K581 residue, located within DDX3X-C (Figure 6K), was most substantially reduced upon METTL3 overexpression, suggesting that METTL3 preferentially targets the region of DDX3X that interacts with it. Collectively, we demonstrated that METTL3 could promote DDX3X expression through inhibiting ubiquitin-proteasome degradation pathway, thereby suppressing the EV71-induced IFN-I response.

## Discussion

METTL3 has been shown to affect innate immunity by catalyzing m6A modifications on viral or host RNAs.^81^^,^^82^ The present study uncovers two distinct pathways by which METTL3 suppresses EV71-induced immune responses. On one hand, deposition of m6A modifications on EV71 by the MT-A70 domain of METTL3 attenuated RIG-I binding to viral RNAs. On the other hand, METTL3 targeted the N-terminus of DDX3X independent of its m6A catalytic motif and stabilized DDX3X by inhibiting its ubiquitination, which subsequently acted as the mediator of the inhibitory effects of METTL3 on IFN-I-related immune responses. This study characterizes a role for METTL3 beyond its function as an m6A writer in regulating immune responses.

In our study, EV71 infection enhanced METTL3 expression in both Vero and RD cells. Notably, many viral infections affect METTL3 expression. For instance, severe acute respiratory syndrome coronavirus 2 (SARS-CoV-2) enhanced METTL3 expression in Vero E6 cells but suppressed METTL3 transcripts in the lung samples from patients.^57^^,^^83^ Moreover, infection with either respiratory syncytial virus (RSV) or vesicular stomatitis virus (VSV) promoted the expression of METTL3,^84^^,^^85^ whereas infection with Japanese encephalitis virus (JEV) inhibited METTL3 expression in mouse brain tissue.^86^ In HIV-1-infected CD4^+^ T cells, only cellular m6A-modified RNA levels were increased, and the expression of METTL3 was not affected.^87^ These researches displayed different expression patterns of METTL3 with different viral infections and cell types. In the present study, the regulatory mechanisms of METTL3 expression differed between Vero and RD cells. The transcriptional levels of *METTL3* were increased after EV71 infection in Vero cells, whereas in RD cells, EV71 infection inhibited METTL3 ubiquitination but did not alter METTL3 RNA levels. The observed regulation in Vero cells may be attributed to the immunodeficiency of Vero cells, characterized by a lack of IFN-I production, which might in turn inhibit the transcriptional expression of METTL3. Coincidently, in lung samples collected from patients infected with SARS-CoV-2, the expression of most inflammatory genes and interferon-stimulated genes (ISGs) was increased, and the levels of METTL3 transcripts were significantly downregulated, compared to those in samples from healthy individuals.^83^ In contrast, a recent study showed that SARS-CoV-2 infection could enhance METTL3 expression in Vero cells.^57^ These discrepancies highlight the potential impact of IFN on METTL3 expression. Moreover, METTL3 could promote SARS-CoV-2 replication in Vero cells but exhibited opposite effects in Huh7 cells.^57^^,^^88^ Overall, these results indicate that the mechanisms governing METTL3 regulation, as elucidated in this study, contribute to resolving the previously observed inconsistencies in the regulation of METTL3 in immunodeficient cells versus normal cells described in earlier studies.

The catalytic center of METTL3 inhibits immune responses by attenuating the RNA affinity to RIG-I. METTL3 and m6A have been reported to promote EV71 replication.^61^^,^^62^^,^^63^ In this study, METTL3-mediated m6A was also shown to inhibit EV71-induced immune responses through suppressing the binding of RIG-I. This phenomenon is similar to that observed in SARS-CoV-2, RSV, HBV, HCV, and VSV infections, where m6A also assists these viral RNAs in evading recognition by RIG-I.^83^^,^^89^^,^^90^^,^^91^ In previous studies, the detailed mechanisms by which m6A inhibits RIG-I recognition varied across different viruses. For instance, the m6A modification inhibits the recognition of VSV RNA by RIG-I through inhibiting the formation of double-stranded RNA.^91^ In HBV and HCV, m6A inhibits RIG-I recognition by recruiting YTHDF2 and YTHDF3.^90^ The specific mechanism by which m6A inhibits RIG-I’s recognition of EV71 RNA is being investigated. Given that the m6A modification can inhibit the recognition of invading RNAs by both TLR and RIG-I^35^^,^^36^ and that TLR is also an important PRR in EV71-mediated immune response,^59^ it is possible that the m6A on EV71 RNAs may also affect the functionality of TLR. However, this hypothesis requires further investigation.

In the present study, it was the overexpression of ΔMT-A70 region of METTL3, rather than the MT-A70, that inhibited EV71-induced immune response. This is likely due to the ΔMT-A70 region of METTL3 interacting with DDX3X to promote DDX3X stability, thereby dampening the expression of IFNA and IFNB. Meanwhile, we noted that METTL3-mediated EV71 m6A could inhibit IFN-I response. This inconsistency is likely due to MT-A70 missing the N-terminus of METTL3 required for WTAP binding,^92^^,^^93^ which affected the assembly of the methyltransferase complex, resulting in the failure of EV71 m6A modification. Besides, we found that the m6A-mut1 EV71 enhanced the immune responses more significantly than m6A-mut2 and exhibited a stronger affinity to RIG-I. This result confirms the function of RIG-I in the regulation of the immune responses by m6A and suggests that the location of m6A modifications in the EV71 genome may have varying effects on immune responses.

An increasing number of studies show that m6A methyltransferase can function in an m6A-independent manner. Cytoplasm-anchored METTL3 interacts with PABPC1 to stabilize its association with eIF4F, thus prioritizing the translation of epigenetic factors without m6A modification.^94^ METTL3 and METTL14 transcriptionally drive the senescence-associated secretory phenotype (SASP) in an m6A-independent manner.^95^ In the nucleus, METTL16 functions as an m6A writer, whereas in the cytosol, METTL16 boosts translation in an m6A-independent manner.^96^ In the cytoplasm, both METTL3 and METTL16 promote translation in an m6A-independent manner.^94^^,^^96^ EV71 infection promotes the distribution of METTL3 in the cytoplasm,^61^ suggesting that the effect of METTL3 on immune responses through an m6A-independent manner may also require its re-localization, which requires further verification.

In the present study, METTL3 interacted with DDX3X independently of RNA and enhanced DDX3X expression by inhibiting its ubiquitination. Previously, we reported that METTL3 can promote the expression of EV71 RNA-dependent RNA polymerase (3D) by affecting 3D ubiquitination modifications.^61^ This effect may be attributed to the interaction between METTL3 and many ubiquitin-related factors during EV71 infection, thereby regulating the formation of ubiquitination in DDX3X and 3D.

During viral infections, DDX3X enhances the production of IFN by interacting with TANK-binding kinase 1 (TBK1) and inhibitor of nuclear factor kappa-B kinase ε (IKKε) to augment IRF signaling or sensing viral sense viral RNA and to supplement the function of RIG-I and MDA-5 in the early phase of infection.^42^^,^^72^^,^^73^^,^^74^^,^^97^ On the contrary, DDX3X was found to inhibit EV71-induced antiviral innate immunity. Similar to our observation, DDX3X has been shown to interact with the NF-κB subunit p65 and suppress NF-κB-mediated transcriptional activity.^76^ Moreover, DDX3X depletion triggers a tumor intrinsic IFN-I response in breast cancer cells.^77^ These data demonstrate the complex functions of DDX3X in immune response, suggesting that it may play opposite roles in different cells and viral infections.

In conclusion, this study revealed an important regulatory function of the METTL3 in antiviral innate immunity. With the detailed mechanisms now elucidated, future studies could focus on the underlying reasons for the effect of METTL3 on DDX3X ubiquitination and unravel the mechanisms by which DDX3X inhibits EV71-induced antiviral immunity. Although the present study identified DDX3X as a promising antiviral target, current inhibitors have a major impact on the overall host RNA metabolism. Given that METTL3 targeted DDX3X for binding and regulated its expression, manipulation of DDX3X could be achieved by interfering with METTL3 expression at the transcriptional or post-translational levels. Our study not only expands the functional repertoire of METTL3 beyond m6A modifications but also provides additional insights and potential drug targets for developing antiviral drugs.

### Limitations of the study

Although our study has demonstrated the dual role of METTL3 in inhibiting EV71-induced immune responses, several issues remain unsolved. First, our finding that METTL3 suppresses antiviral immune responses via both m6A-dependent and -independent pathways was derived from *in vitro* studies and have yet to be validated *in vivo* using a METTL3-deficient mouse model. This is mainly because of the extremely low infection efficiency of the EV71-XF strain in immunocompetent mice, and further studies should consider the introduction of other mouse-adapted EV71 strains. Second, although certain lysine sites in DDX3X have been identified as potentially involved in METTL3-mediated ubiquitination, the exact mechanism by which METTL3 regulates DDX3X ubiquitination remains to be investigated. Third, our study showed that MDA5 may be implicated in DDX3X-mediated immune inhibition during EV71 infection, but the specific molecular mechanisms underlying METTL3-DDX3X-MDA5 axis have not been thoroughly explored and needs further exploration.

## Resource availability

### Lead contact

Further information and requests for resources and reagents should be directed to and will be fulfilled by the lead contact, Wuxiang Guan (guanwx@wh.iov.cn).

### Materials availability

Plasmids and the viruses used in this study can be obtained from the lead contact after the permission of original distributors.

### Data and code availability

• All data reported in this paper will be shared by the lead contact upon request.
• This paper does not report original code.
• Any additional information required to reanalyze the data reported in this paper is available from the lead contact upon request.

## Acknowledgments

We thank Dr. Bo Zhang for providing the EV71 infectious clone and Dr. Hanzhong Wang for providing ubiquitination related plasmids. We are grateful to Dr. Hongyan Wang for providing iBMDMs. This work was supported by Strategic Priority Research Program of the Chinese Academy of Sciences [XDB0490000], Wuhan Knowledge Innovation Special Project [2023020201020303], 10.13039/501100003819Natural Science Foundation of Hubei Province [2024AFB1065], 10.13039/501100001809National Natural Science Foundation of China [31970168], Hubei Jiangxia Laboratory Biosafety Key R&D Project [JXBS017, JXBS013], and Hubei Jiangxia Laboratory Research Startup Funding Project [E3ZFJX0101].

## Author contributions

W.G. and H.H. conceptualized and designed the research. H.H. and F.Z. performed the majority experiments and statistical analysis. Z.C., Z.T., H.Z., X.F., X.Z., T.D., G.Z., T.L., K.Z., S.D., and H.L. participated in part of the experiments. Z.Z. and Y.W. provided experimental design and guidance. H.H. wrote the manuscript. F.H. and F.Z. helped to revise the manuscript. H.H. and F.Z. contributed equally to this work.

## Declaration of interests

The authors declare no competing interests.

## STAR★Methods

### Key resources table

**Table:** 

REAGENT or RESOURCE	SOURCE	IDENTIFIER
**Antibodies**		

Rabbit monoclonal anti-METTL3	Proteintech	Cat# 15073-1-AP; RRID:AB_2142033
Mouse monoclonal anti-GAPDH	Proteintech	Cat# 60004-1-Ig; RRID:AB_2107436
Rabbit polyclonal anti-VP1	GeneTex	Cat# GTX132339; RRID:AB_2886617
Mouse monoclonal anti-β-actin	Santa Cruz Biotechnology	Cat# sc-47778; RRID:AB_626632
Rabbit monoclonal anti-p-STAT1	Cell Signaling Technology	Cat# 9167; RRID:AB_561284
Rabbit polyclonal anti-p-STAT2	Cell Signaling Technology	Cat# 4441; RRID:AB_2198445
Rabbit polyclonal anti-DDX3X	Proteintech	Cat# 11115-1-AP; RRID:AB_10896499
Mouse monoclonal anti-Flag	Sigma-Aldrich	Cat# F1804; RRID:AB_262044
Mouse monoclonal anti-HA	Proteintech	Cat# 66006-1-Ig; RRID:AB_2857911
Rabbit monoclonal anti-*p*-TBK1	Cell Signaling Technology	Cat# 5483; RRID:AB_10693472
Rabbit monoclonal anti-TBK1	Cell Signaling Technology	Cat# 3504; RRID:AB_2255663
Rabbit monoclonal anti-*p*-IRF3	Cell Signaling Technology	Cat# 4947; RRID:AB_823547
Rabbit monoclonal anti-IRF3	Cell Signaling Technology	Cat# 4302; RRID:AB_1904036
Rabbit polyclonal anti-ISG15	Proteintech	Cat# 15981-1-AP; RRID:AB_2126302
Rabbit polyclonal anti-IFIT1	Proteintech	Cat# 23247-1-AP; RRID:AB_2811269
Rabbit polyclonal anti-p65	Proteintech	Cat# 10745-1-AP; RRID:AB_2178878
Rabbit polyclonal anti-IFIH1/MDA5	Proteintech	Cat# 21775-1-AP; RRID:AB_10734593
Rabbit monoclonal anti-RIG-I	Cell Signaling Technology	Cat# 3743; RRID:AB_2269233
Rabbit IgG	Proteintech	Cat# 30000-0-AP; RRID:AB_2819035
Mouse IgG	Proteintech	Cat# B900620; RRID:AB_2883054

**Bacterial and virus strains**		

Enterovirus 71 (EV71,strain XF)	Microorganisms & Viruses Culture Collection Center, Wuhan Institute of Virology (WIV), Chinese Academy of Sciences (CAS)	N/A
WT, m6A-mut EV71	Hao et al.^61^	N/A
Sendai virus (SeV)	Dr. Yanyi Wang, WIV, CAS	N/A

**Chemicals, peptides, and recombinant proteins**		

3-Methyladenine	MedChemExpress	Cat# HY-19312(50mg)
Chloroquine	MedChemExpress	Cat# HY-17589A
MG132	Beyotime	Cat# S1748-5mg
TAK-243 (MLN7243)	Selleck	Cat# 1450833-55-2
NSC697923	Selleck	Cat# 343351-67-7
Actinomycin D	Sigma-Aldrich	Cat# SBR00013-1mL
Cycloheximide	Sigma-Aldrich	Cat# C7698
ATP	Thermo Fisher Scientific	Cat# R0441
m6ATP	TriLink	Cat# *N*-1013
DMRIE-C Reagent	Thermo Fisher Scientific	Cat# 10459014
Lipofectamine 2000 Transfection reagent	Thermo Fisher Scientific	Cat# 11668019
Lipofectamine™ RNAiMAX	Thermo Fisher Scientific	Cat# 13778150
M-MLV reverse transcriptase	Thermo Fisher Scientific	Cat# 28025021
DMSO	Sigma-Aldrich	Cat# D2650
Trizol reagent	Thermo Fisher Scientific	Cat# 15596026CN
Protein A Dynabeads	Thermo Fisher Scientific	Cat# 10002D
PMSF	Sangon Biotech	Cat# A100754
RNAse inhibitor	Promega	Cat# N2515
Protein G agarose beads	Millipore	Cat# 16-266
Protein–A agarose beads	Millipore	Cat# 16-125

**Critical commercial assays**		

Human IFN-α ELISA kit	MEIMIAN	Cat# MM-51340H1
Human IFN-β ELISA kit	MEIMIAN	Cat# MM-51652H1
Mouse IFN-β ELISA kit	BioLegend	Cat# 439407
MEGAscript T7 Transcription Kit	Thermo Fisher Scientific	Cat# AMB13345
Luciferase assay kit	Promega	Cat# E1500
Hieff® qPCR SYBR® Green Master Mix	Yeasen	Cat# 11201ES08

**Experimental models: Cell lines**		

Vero	ATCC	Cat# CCL-81
HEK293T	ATCC	Cat# CRL-11268
HeLa	ATCC	Cat# CCL-2
RD	ATCC	Cat# CCL-136
MDCK	ATCC	Cat# CCL-34
BHK-21	ATCC	Cat# CCL-10
Huh7.5.1	Dr. Xinwen Chen, WIV, CAS	N/A
iBMDMs	Dr. Hongyan Wang, Center for Excellence in Molecular Cell Science, CAS	N/A
PEMs	Isolated from mice	N/A

**Oligonucleotides**		

shRNA targeting sequences (see Table S1)	This paper	N/A
siRNA targeting sequences (see Table S1)	This paper	N/A
Primers for RT-qPCR (see Table S2)	This paper	N/A

**Recombinant DNA**		

pcDNA3.0	Hao et al.^61^	N/A
pUltra	Zhang et al.^98^	N/A
pXJ40-Flag/HA	Hao et al.^61^	N/A
pMETTL3	Hao et al.^61^	N/A
pUltra-METTL3	This paper	N/A
pUltra-METTL3mut	This paper	N/A
pFlag-METTL3	Hao et al.^61^	N/A
pFlag-DDX3X	This paper	N/A
pMETTL3mut	Hao et al.^61^	N/A
pHA-METTL3mut	This paper	N/A
pHA-ΔMT-A70	This paper	N/A
pHA-MT-A70	This paper	N/A
pFlag-DDX3X-N	This paper	N/A
pFlag-DDX3X-C	This paper	N/A
DDX3X-K50R, -K55R, -K64R, -K66R, -K81R, -K130R, -K138R, -K162R, -K208R, -K264R, and -K581R	This paper	N/A
pHA-K6	MIAOLING BIOLOGY	Cat# P51114
pHA-K11	MIAOLING BIOLOGY	Cat# P51118
pHA-K27	MIAOLING BIOLOGY	Cat# P51092
pHA-K33	MIAOLING BIOLOGY	Cat# P51113
pCDNA3.0-HA-MDA5	MIAOLING BIOLOGY	Cat# P2155
pCDNA3.0-HA-p65	This paper	N/A
EV71 infectious clone	Dr. Bo Zhang, WIV, CAS	N/A
pHA-Ubi	Dr. Hanzhong Wang, WIV, CAS	N/A
pHA-K48 and –K63	Dr. Xinwen Chen, WIV, CAS	N/A
IFNB promoter plasmid	Dr. Yanyi Wang, WIV, CAS	N/A
ISRE reporter plasmid	Dr. Yanyi Wang, WIV, CAS	N/A
NF-κB reporter plasmid	Dr. Yanyi Wang, WIV, CAS	N/A
pLKO.1-shNC	Hao et al.^61^	N/A
pLKO.1-shMETTL3-1	This paper	N/A
pLKO.1-shMETTL3-2	This paper	N/A
pSPAX	Hao et al.^61^	N/A
pMD2G	Hao et al.^61^	N/A

**Software and algorithms**		

ImageJ	NIH	https://imagej.nih.gov/ij/
Bio-Rad CFX Maestro 2.3	Bio-Rad	https://www.bio-rad.com/
GraphPad Prism8	GraphPad Prism Inc	https://www.graphpad.com/

### Experimental model and study participant details

#### Cell culture

Vero, HEK293T, HeLa, RD, MDCK and BHK-21 cells were purchased from the ATCC. Huh7.5.1 cells were obtained from the Wuhan Institute of Virology (WIV) of the Chinese Academy of Sciences (CAS). iBMDMs were kindly provided by Dr. Hongyan Wang (Center for Excellence in Molecular Cell Science, CAS). iBMDMs expressing METTL3 and METTL3mut were generated by transfecting HEK293T cells with pUltra-METTL3 and pUltra-METTL3mut plasmids, along with the lentiviral packaging plasmids pSPAX and pMD2G. Lentiviral supernatants were then collected and used to infect iBMDMs, followed by sorting for GFP^+^ cell. PEMs were harvested from mice with intraperitoneal injection of 3% brewer thioglycollate medium (3 mL) for 4 days. All cells were cultured in Dulbecco’s modified Eagle’s medium (DMEM; Gibco, Gaithersburg, MD, USA) supplemented with 10% fetal bovine serum (Gibco) and 1% penicillin/streptomycin (100 U/mL) at 37°C with 5% CO2.

#### Virus

EV71 (strain XF) was obtained from the Microorganisms & Viruses Culture Collection Center, WIV, CAS. WT, m6A-mut EV71 were rescued from infectious DNA constructs in our laboratory. SeV were provided by Dr Yanyi Wang (WIV, CAS). All viruses were titrated using 50% tissue culture infectious dose (TCID50) assays with the Reed-Muench formula.^99^

#### Mice

6-week-old female or male C57BL/6 mice were purchased from GemPharmatech Co.,Ltd. Mice for PEMs preparation were conducted in strict accordance with the institutional guidelines and were approved by the Ethical Committee for Animal Experiments of Wuhan Institute of Virology, Chinese Academy of Sciences (Approval No. WIVA32202303).

### Method details

#### Plasmid construction

pMETTL3, pUltra-METTL3, pUltra-METTL3mut, pFlag-METTL3 and pFlag-DDX3X plasmids were constructed by inserting the CDS sequence of METTL3 (GenBank: NM_019852.5) or DDX3X (GenBank: NM_001356.5) from Homo sapiens into pcDNA3.0, pUltra,^98^ or pXJ40-Flag. The METTL3 CDS region was mutated and truncated accordingly and inserted into pcDNA3.0 or pXJ40-HA to construct the pMETTL3mut, pHA-METTL3mut, pHA-ΔMT-A70, and pHA-MT-A70 plasmids. The DDX3X CDS was truncated and inserted into pXJ40-Flag to generate the pFlag-DDX3X-N and pFlag-DDX3X-C plasmids. DDX3X-K50R, -K55R, -K64R, -K66R, -K81R, -K130R, -K138R, -K162R, -K208R, -K264R, and -K581R were constructed by mutating corresponding bases on pFlag-DDX3X. pHA-K6, -K11, -K27, -K33, and pCDNA3.0-HA-MDA5 were purchased from MIAOLING BIOLOGY. pCDNA3.0-HA-p65 was constructed by inserting the CDS sequence of p65 from Homo sapiens into pcDNA3.0. EV71 infectious clone and pHA-Ubi plasmid were provided by Dr. Bo Zhang and Dr. Hanzhong Wang (WIV, CAS), respectively. pHA-K48 and –K63 were gifted by Dr. Xinwen Chen (WIV, CAS). Expression plasmids for the *IFNB* promoter, ISRE and NF-κB reporter were provided by Dr. Yanyi Wang (WIV, CAS).

#### ELISA

Concentrations of IFN-β and IFN-α in cell culture supernatants were measured by Human IFN-α ELISA kit (MM-51340H1, MEIMIAN), Human IFN-β ELISA kit (MM-51652H1, MEIMIAN), or Mouse IFN-β ELISA kit (439407, BioLegend) according to the manufacturer’s instructions.

#### Western blot analysis

Various cells, including EV71-infected or IFN-β-treated cells were harvested and lysed at the indicated times. Cell lysates were separated by SDS polyacrylamide gel electrophoresis and transferred onto nitrocellulose membranes. Subsequently, the nitrocellulose membranes were blocked with 5% non-fat milk and then incubated with the designated antibodies shown in the key resources table at 4°C overnight. The membranes were washed three times with TBST buffer and incubated with secondary antibodies for 1 h at room temperature. Afterward, the membranes were visualized using enhanced chemiluminescence reagents, and the signals were captured by Chemiluminescence Imaging system (Tanon 4800).

#### qRT-PCR

RNA was extracted using TRIzol reagent (Invitrogen) and reverse transcribed using M-MLV reverse transcriptase (Invitrogen). qRT-PCR was performed using Hieff qPCR SYBR Green Master Mix (Yeasen) on a CFX Connect Real-Time system (Bio-Rad). All the gene expression levels were obtained using the $2^{-\Delta \Delta \text{Cq}}$ method with Bio-Rad CFX Maestro 2.3. The primers used in qRT-PCR were shown in Table S2.

#### Quantification of ribosome-loaded RNA

HeLa, RD, HEK293T, MDCK, Vero, BHK, and Huh7.5.1 cells were seeded for 18 h and treated with 100 μg/mL CHX for 10 min at 37°C. The cells were washed three times by PBS and lysed in ribosome lysis buffer (10 mM Tris-HCl, pH 7.4, 100 mM KCl, 5 mM MgCl_2_, 1% Triton X-100, 2 mM DTT, 100 U/mL RNase inhibitor, 1 μg/mL proteinase inhibitor, and 100 μg/mL CHX). The lysates clarified by centrifugation at 2,000 × *g* for 10 min at 4°C. One-tenth of the supernatant was used as the input sample. The remaining supernatant was loaded onto a 10–45% sucrose gradient and centrifuged at 30,000 × *g* for 3 h at 4°C. RNAs were extracted from the input sample or ribosomal pellet using TRIzol and quantified by qRT-PCR.

#### Ubiquitination assays

RD, Vero or HEK293T cells were co-transfected with the indicated plasmids for 18 h and then infected with mock or EV71 for 12 h. The cells were lysed and centrifuged at 14 000 rpm at 4°C for 10 min. Simultaneously, 10 μg of anti-Flag antibody was incubated with 50 μL of protein A Dynabeads for 20 min, followed by incubation with the cell lysates for 30 min. The complexes were washed six times with PBST (PBS with 0.02% Tween 20) and detected using western blotting.

#### Transfection and reporter assays

Cells were seeded in 48-well plates and transfected by standard calcium phosphate precipitation with the indicated plasmids, and 0.01 μg of pRL-TK (*Renilla* luciferase) reporter plasmid was added to normalize for transfection efficiency. Simultaneously, an empty control plasmid was added to equalize the amount of total plasmid DNA in each transfection. After 24 h of transfection, cells were treated with the indicated stimuli. Luciferase assays were performed using a dual-specific luciferase assay kit (Promega).

#### shRNA and siRNA-mediated gene silencing

The shRNA target sequences for human *METTL3* and *DDX3X* were provided in Table S1. The shRNAs were cloned into pLKO.1-TRC and packaged into lentiviruses using psPAX2 and pMD2.G according to the manufacturer’s instructions. Stable RD or HEK293T knockdown cell lines were screened using 2 μg/mL puromycin after lentiviral infection.

The siRNAs targeting *DDX3X*, *P65*, *MDA5*, and *Mettl3* were listed in Table S1 siRNAs (40 nM) were transfected into cells using Lipofectamine RNAiMAX reagent (Invitrogen) for 48 h. Total RNA was extracted with TRIzol reagent, and cDNA was obtained with reverse transcriptase M-MLV for qRT-PCR.

#### The transfection of EV71 genome

EV71 genomic RNAs were *in vitro* transcribed from a HindIII (ThermoFisher)-linearized infectious DNA construct with ATP (ThermoFisher) or m6ATP (TriLink, San Diego, CA) as substrates using a MEGAscript T7 Kit (Ambion, Austin, TX, USA) and the RNAs were transfected into HEK293T cells using DMRIE-C Reagent (ThermoFisher).

#### Formaldehyde-crosslinked RNA-immunoprecipitation (RIP) and qRT-PCR

m6A(±)-T7-EV71-transfected or WT and m6A-mut EV71-infected HEK293T or RD cells were cross-linked by phosphate buffered saline (PBS) containing 1% methanol-free formaldehyde and incubated for 10 min at 37°C. Subsequently, the crosslinking was terminated by 0.125 M glycine and the cells were washed with PBS and lysed using RIP buffer (150 mM KCl, 25 mM Tris-HCl pH 7.4, 5 mM EDTA, 0.5 mM dithiothreitol [DTT], 0.5% NP40, 100 U/ml RNase inhibitor, 100 μM phenylmethylsulfonyl fluoride [PMSF], and 1 μg/mL proteinase Inhibitors) for 30 min. The lysates were centrifuged, and the supernatants were incubated with antibodies against RIG-I (Cell Signaling Technology) overnight at 4°C. The mixtures were then incubated with 30 μL preblocked protein-A agarose beads for 2 h at 4°C and washed thrice with the washing buffer (300 mM KCl, 25 mM Tris-HCl pH 7.4, 5 mM EDTA, 0.5 mM DTT, 0.5% NP40, 100 U/ml RNase inhibitor, 100 μM PMSF, and 1 μg/mL proteinase Inhibitors), followed by three washes using 1 mL RIP buffer. RNA was extracted using TRIzol reagent and quantified by qRT-PCR.

#### Co-IP and immunoblot analysis

HEK293T cells co-transfected with indicated plasmids were lysed in 1.2 mL IP buffer (50 mM Tris [pH = 7.5], 1 mM EGTA, 1mM EDTA, 1% Triton X-100, 150 mM NaCl, 2 mM DTT, 100 μM PMSF, and 10 μg/mL leupeptin). For each IP, 0.5 mL cell lysate was incubated with IgG (0.5 μg) or the indicated antibody (0.5 μg) at 4°C for 3 h, and 15 μL of pre-blocked protein-G or -A agarose beads were added to each sample for 1 h at 4°C. The beads were washed six times using an IP buffer containing 0.3 M NaCl. The proteins were analyzed by western blotting.

### Quantification and statistical analysis

All sample sizes were large enough to ensure proper statistical analysis. Data were analyzed by GraphPad Prism Software (La Jolla, CA, USA), and details of the statistical tests used were specified within the corresponding figure legends. Data are presented as the means ± standard deviations (SDs) or ±standard error of the mean (SEM) (*n* = 3). ns, no significance (*p* > 0.05). *p* value ≤0.05 was considered statistically significant.
