# Supplementary material for: Distinct pathways utilized by METTL3 to regulate antiviral innate immune response
Source: iScience. 2024 Sep 30;27(11):111071. doi: 10.1016/j.isci.2024.111071 (PMC11700651; doi:10.1016/j.isci.2024.111071)
Supplement: Document S1. Figures S1–S8, Tables S1, and S2 [file mmc1.pdf]

iScience, Volume *27*

Supplemental information

Distinct pathways utilized by METTL3 to regulate antiviral innate immune response

Haojie Hao, Fang Zhang, Zhen Chen, Zhongyuan Tan, Hongyan Zhang, Xumei Feng, Xueyan Zhang, Tao Deng, Guanli Zhan, Ting Luo, Kui Zhang, Shuang Ding, Haibin Liu, Zhenhua Zheng, Yanyi Wang, Fang Huang, and Wuxiang Guan

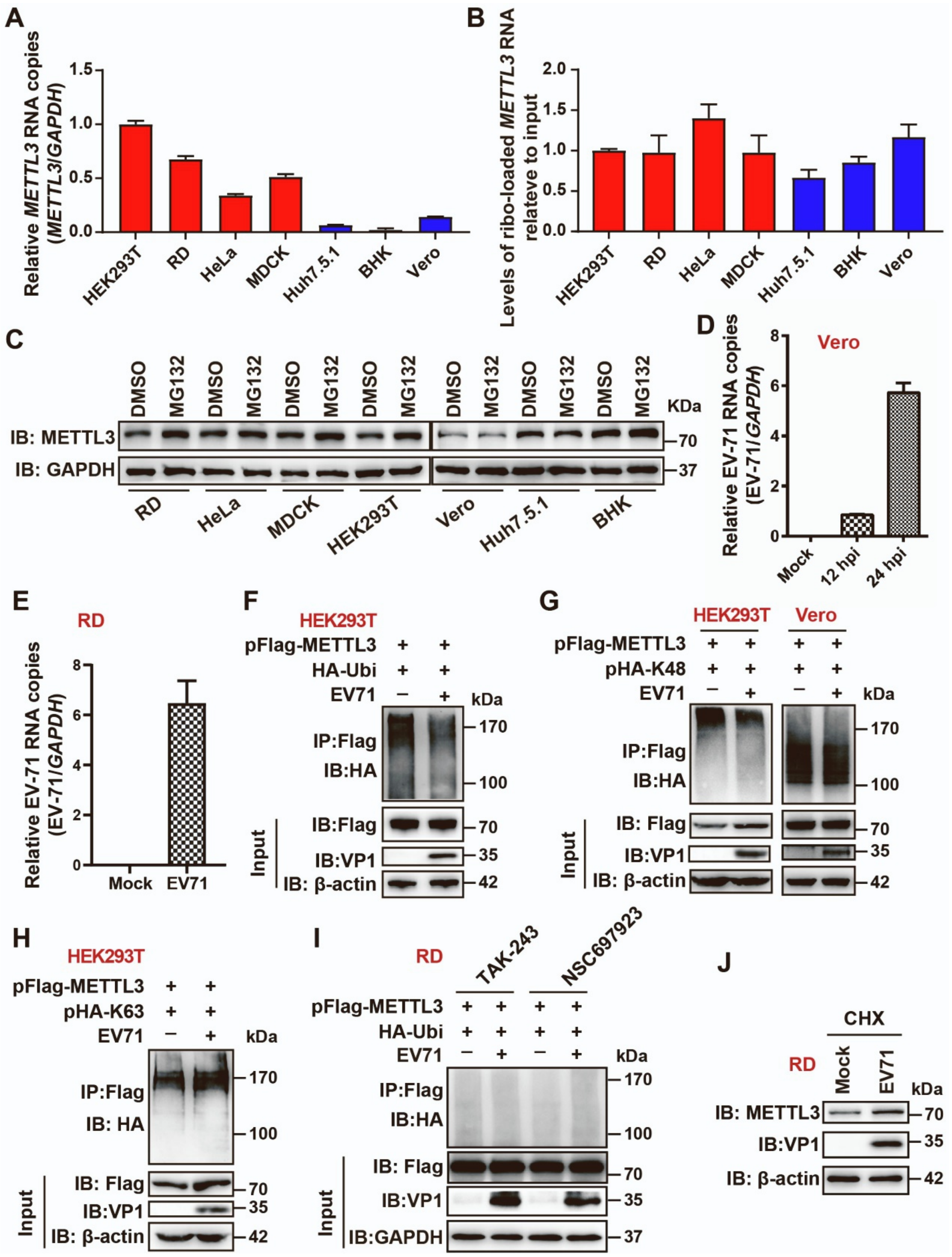

Figure S1. EV71 infection alters METTL3 ubiquitination. Related to Figure 1. (**A**) HEK293T, RD, HeLa, MDCK, Vero, BHK, and Huh7.5.1 cells were seeded and cultured for 18 h and then measured the transcripts of METTL3 by qRT-PCR. Data are means ± SEMs (n = 3). (**B**) The ribosome-loaded METTL3 RNAs in various cells in (A) were detected by sucrose density gradient centrifugation and qRT-PCR. Data are means ± SEMs (n = 3). (**C**) Various cells were treated with DMSO or MG132 and the expression of METTL3 were detected by immunoblot. (**D** & **E**) Total RNA was extracted at the indicated times from EV71-infected Vero (D) and RD (E) cells (MOI = 1). EV71 RNAs were quantified by qRT-PCR, with *GAPDH* used

as a control. Unpaired Student's *t*-test was performed and data are presented as the means ± SEMs (*n* = 3). (**F**-**H**) Ubiquitination assay: HEK293T or Vero cells were transfected with pFlag-METTL3 and pHA-Ub (F), pHA-K48 (G), or pHA-K63 (H), followed by EV71 infection (MOI = 10). IP and immunoblot analysis were performed using the indicated antibodies. (**I**) RD cells were co-transfected with pHA-Ubi and either pFlag-METTL3 or a control vector, followed by EV71 infection. The cells were then treated with TAK-243 and NSC697923 to assess the ubiquitination of METTL3. (**J**) Mock or EV71-infected RD cells were treated with CHX for 6 h. METTL3 expression was detected by immunoblot.

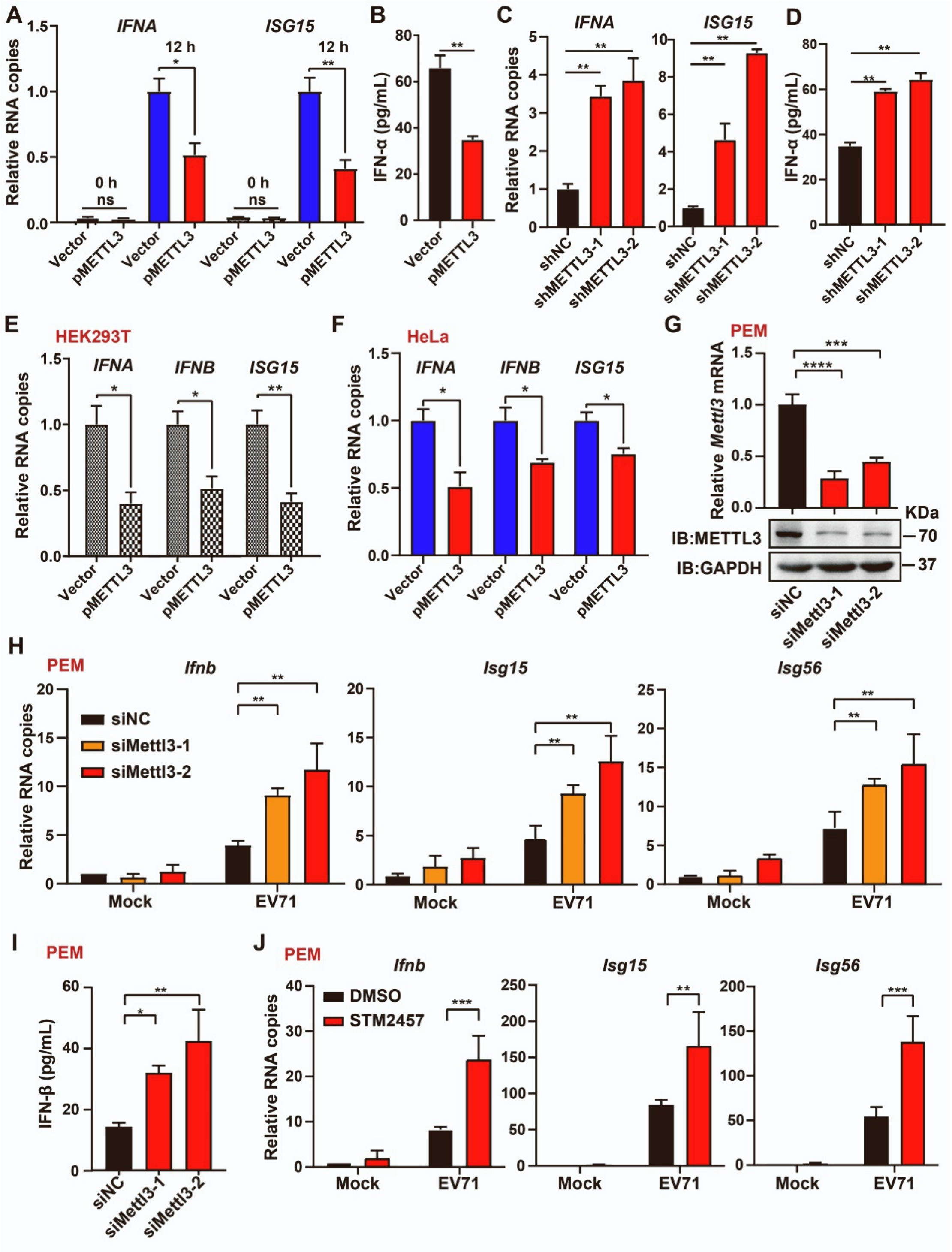

Figure S2. METTL3 and METTL3mut suppress EV71-induced immune response. Related to Figure 2. (**A**-**D**) RD cells were treated with pMETTL3 or shRNA, followed by EV71 infection (MOI = 1). The transcripts of *IFNA* and *ISG15* were detected by qRT-PCR and the concentrations of IFN-α in cell culture supernatants were examined by ELISA. Data are means ± SEMs (n = 3). **P* ≤ 0.05, ***P* ≤ 0.01, ns: not significant, unpaired Student's *t*-tests. (**E** & **F**) HEK293T and HeLa cells were infected with EV71 (MOI = 10 and 5, respectively) and the RNA levels of *IFNA*, *IFNB*, and *ISG15* were quantified by qRT-PCR. Data are means ± SEMs (n = 3). **P* ≤ 0.05, ***P* ⩽ 0.01, unpaired Student's *t*-tests. (**G**) PEMs were transfected with *Mettl3* siRNA and METTL3 expression were detected by qRT-PCR and Western blot. qRT-PCR data are means ± SEMs (n = 3). ****P* ≤ 0.001, *****P* ≤ 0.0001, unpaired Student's *t*-tests. (**H**-**J**) siMettl3- or STM2457-treated PEMs were infected with EV71 and the RNA levels of *Ifnb*, *Isg15* and *Isg56* were quantified by qRT-PCR (H, J). The concentrations of IFN-β in cell culture supernatants were examined by ELISA (I). Data are means ± SEMs (n = 3). **P* ≤ 0.05, ***P* ≤ 0.01, ****P* ≤ 0.001, unpaired Student's *t*-tests (I), or two-way ANOVA with Holm-Sidak's multiple comparisons test (H, J).

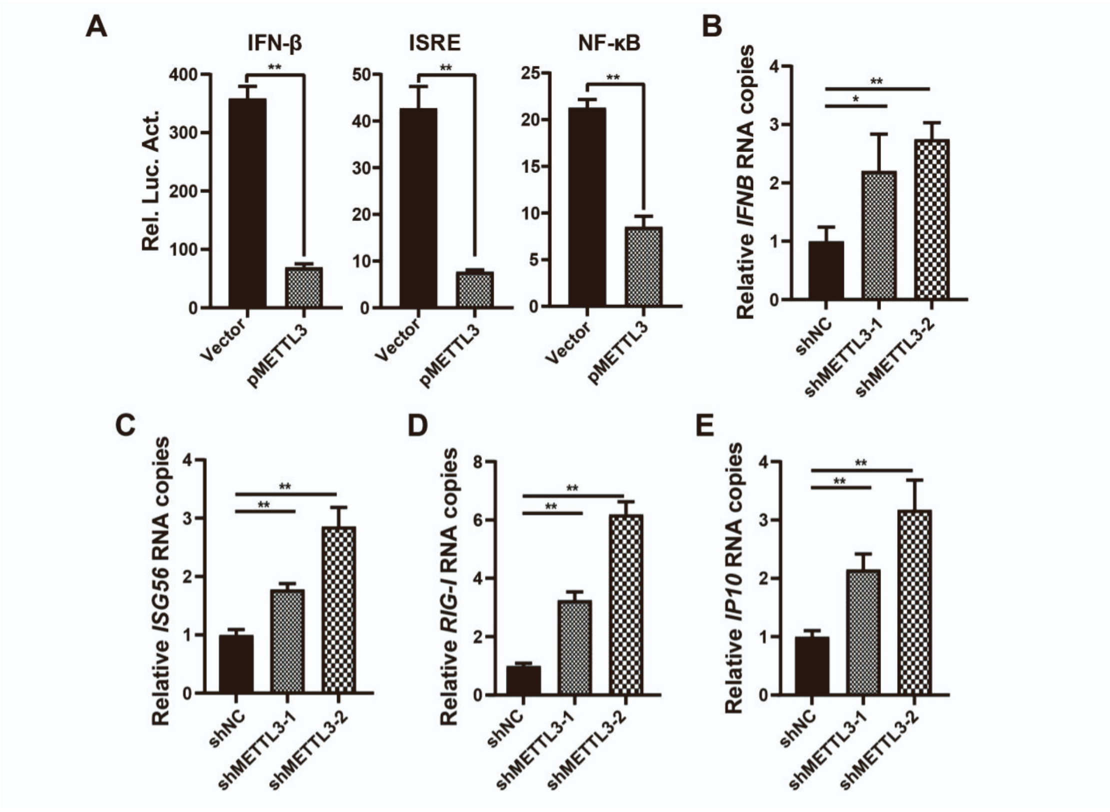

Figure S3. METTL3 suppresses SeV-induced immune response. Related to Figure 2. (**A**) HEK293T cells were transfected with IFN-β, ISRE or NF-κB reporter plasmids plus pMETTL3 or empty vector. After 24 hours, cells were infected with SeV for 12 h, followed by luciferase assays. Data are presented as means ± SDs (n = 3). ***P* ⩽ 0.01, unpaired Student's *t*-tests. (**B**-**E**) shMETTL3 treated-HEK293T cells were infected with SeV for 12 h and total RNAs were extracted to detect the RNA levels of *IFNB* (B), *ISG56* (C), *RIG-I* (D), and *IP10* (E). Data are means ± SEMs (n = 3). **P* ≤ 0.05, ***P* ≤ 0.01, unpaired Student's *t*-tests.

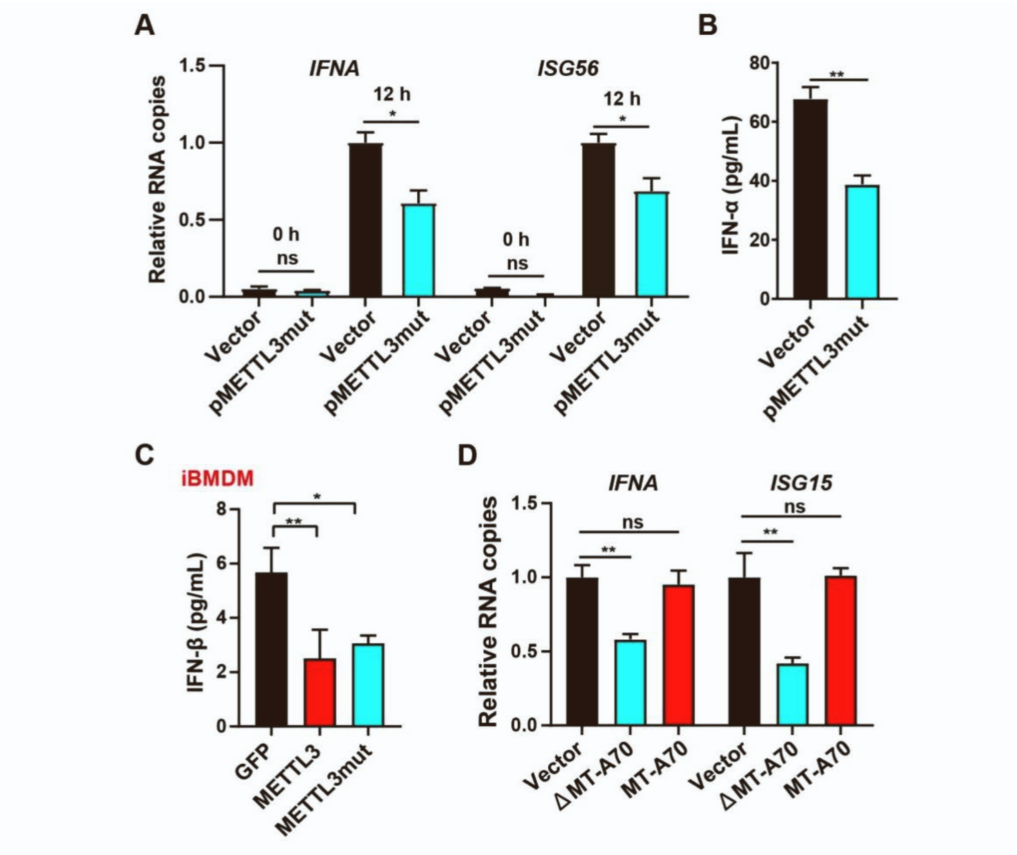

Figure S4. METTL3mut suppresses immune response. Related to Figure 2. (**A** & **B**) Vector or pMETTL3mut transfected-RD cells were infected with EV71. The transcripts of *IFNA* and *ISG56* were detected by qRT-PCR (A) and the concentrations of IFN-α in cell culture supernatants were examined by ELISA (B). Data are means ± SEMs (n = 3). **P* ≤ 0.05, ***P* ≤ 0.01, ns: not significant, unpaired Student's *t*-tests. (**C**) METTL3 and METTL3mut stably overexpressed iBMDMs were infected with EV71, the concentrations of IFN-β in cell culture supernatants were detected by ELISA. Data are means ± SEMs (n = 3). **P* ≤ 0.05, ***P* ≤ 0.01, unpaired Student's *t*-tests. (**D**) Vector, p△MT-A70 and pMT-A70 transfected-HEK293T cells were infected with EV71 and total RNAs were extracted to detect the transcription of *IFNA* and *ISG15*. Data are means ± SEMs (n = 3). ***P* ≤ 0.01, ns: not significant, unpaired Student's *t*-tests.

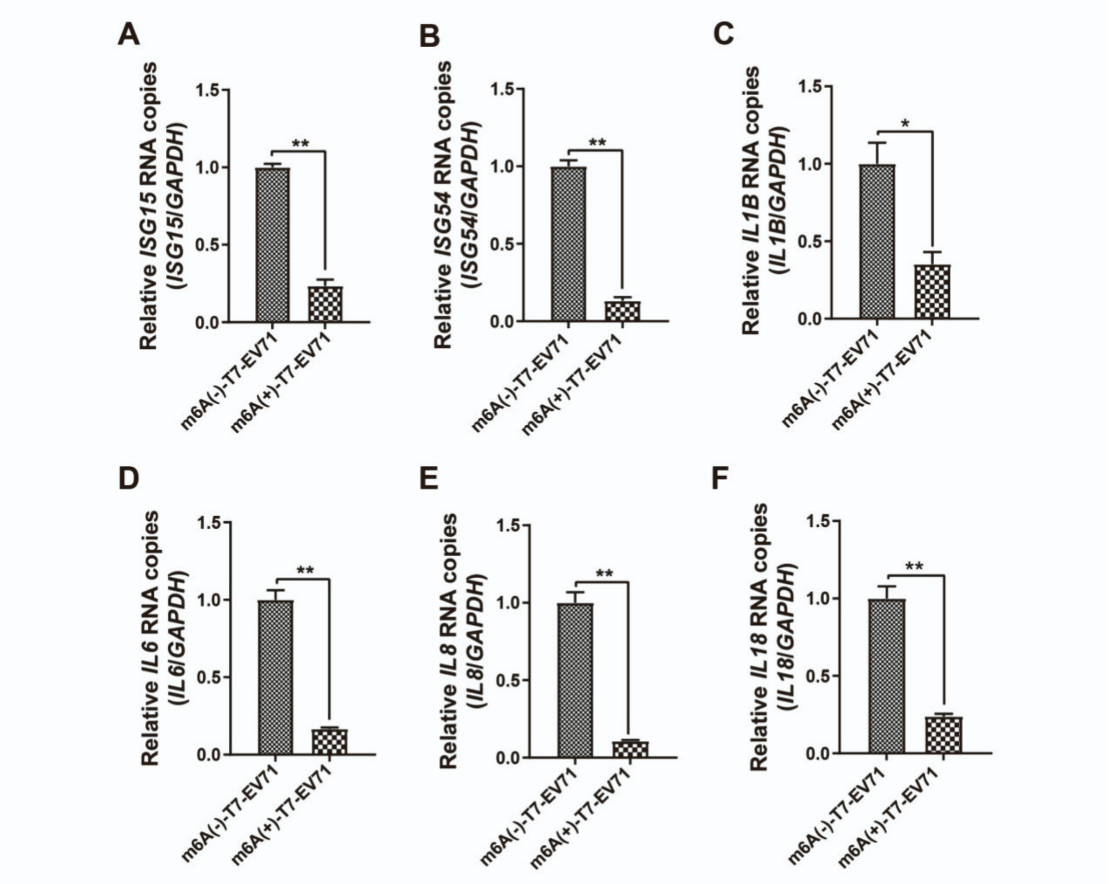

Figure S5. m6A modification on EV71 RNA affects immune response. Related to Figure 3. (**A**-**F**) Total RNA of m6A(±) T7-EV71-transfected HEK293T cells were extracted at 24 h post transfection. The RNA levels of *ISG15* (A), *ISG54* (B), *IL1B* (C), *IL6* (D), *IL8* (E), and *IL18* (F) were quantified by qRT-PCR. Data are means ± SEMs (n = 3). **P* ≤ 0.05, ***P* ≤ 0.01, ns: not significant, unpaired Student's *t*-tests.

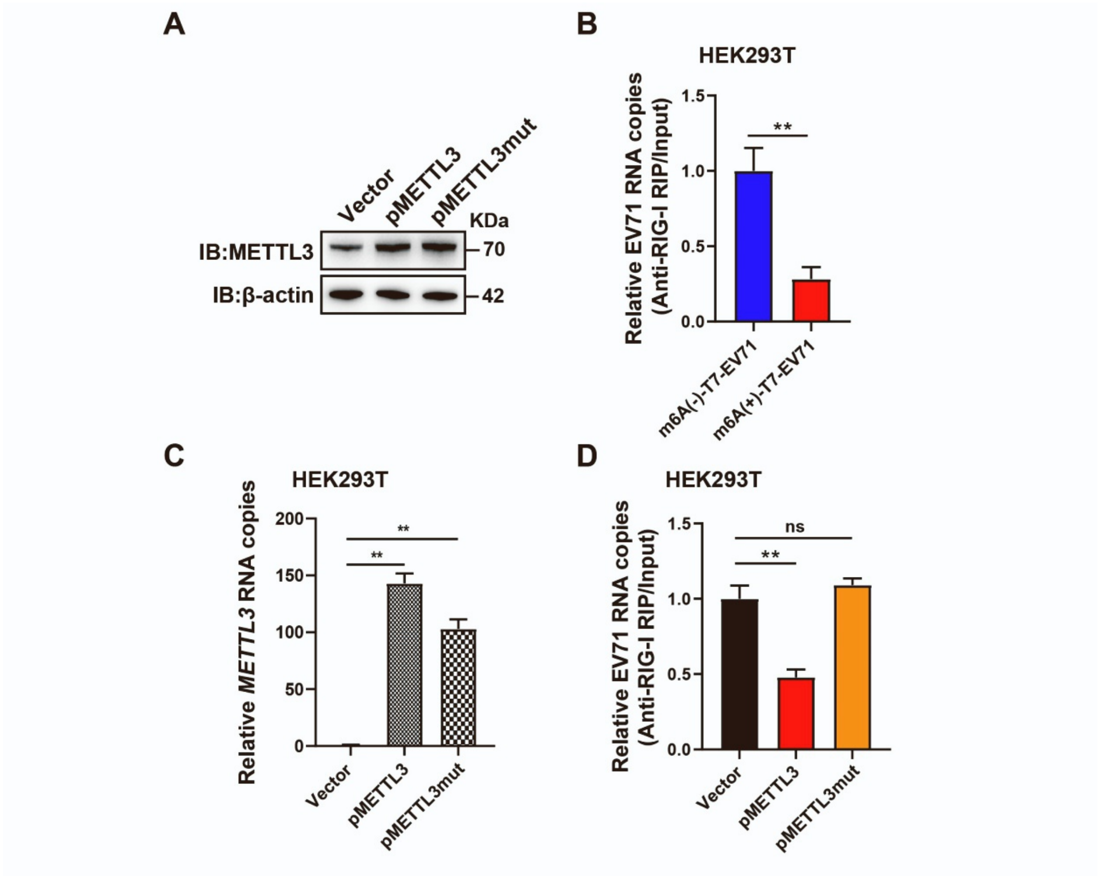

Figure S6. METTL3 inhibits EV71-induced immune response by suppressing RIG-I binding. Related to Figure 3. (**A** & **C**) Vector-, pMETTL3-, or pMETTL3mut-transfected RD (A) or HEK293T (C) cells were infected with EV71 and the expression of METTL3 were detected by Western blot and qRT-PCR. Data are means ± SEMs (n = 3). ***P* ≤ 0.01, unpaired Student's *t*-test. (**B**) m6A affected RIG-I binding in HEK293T cells. m6A(±) T7-EV71 transfected-HEK293T cells were crosslinked with formaldehyde and immunoprecipitated using anti-RIG-I antibodies, followed by qRT-PCR. Data are means ± SEMs (n = 3). ***P* ≤ 0.01, unpaired Student's *t*-test. (**D**) Vector, pMETTL3 or pMETTL3mut transfected-HEK293T cells were infected with EV71 (MOI = 10) and then subjected to the same experiment as (B). Data are means ± SEMs (n = 3). ***P* ≤ 0.01, ns: not significant, unpaired Student's *t*-test.

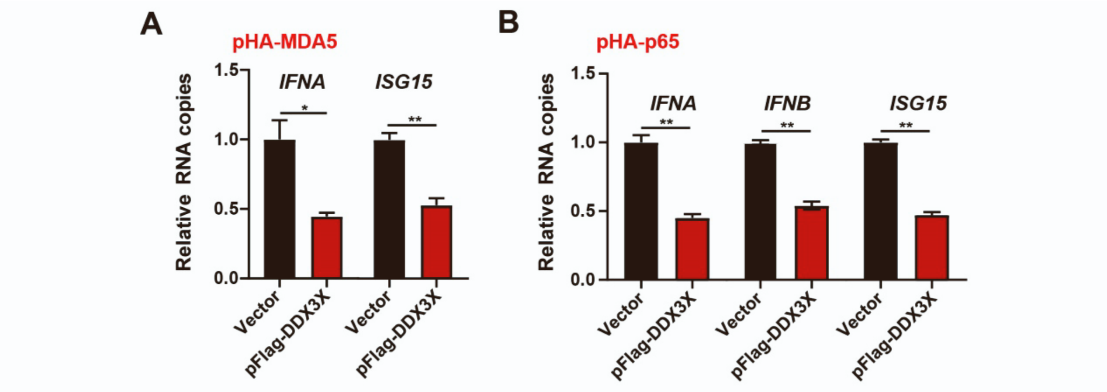

Figure S7. Effects of MDA5 and p65 on DDX3X regulation of EV71-induced immune response. Related to Figure 5. (**A** & **B**) pHA-MDA5 or pHA-p65 was co-transfected with Vector or pFlag-DDX3X into RD cells, followed by EV71 infection. The transcripts of *IFNA*, *IFNB* and *ISG15* were detected using qRT-PCR. Data are means ± SEMs (n = 3). *$P \leq 0.05$, **$P \leq 0.01$, unpaired Student's *t*-tests.

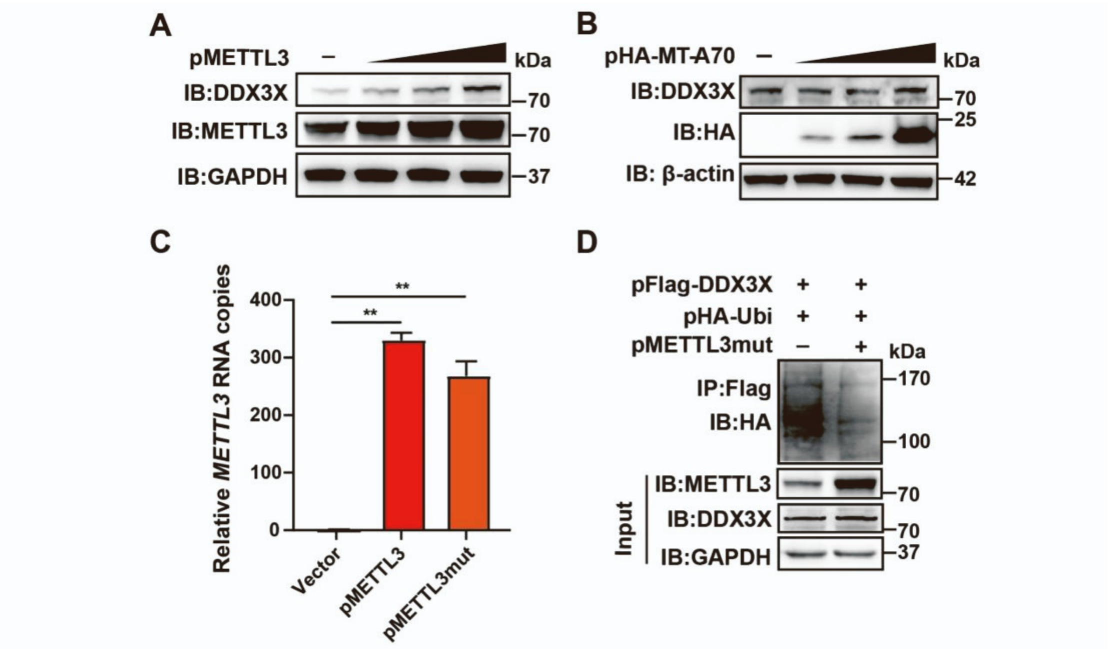

Figure S8. METTL3 promotes DDX3X expression and inhibits its ubiquitination. Related to Figure 6. (**A**) RD cells were transfected with pMETTL3 (0, 0.5, 1, and 2 μg, respectively) in 6-well plates, and missing plasmids in each well were replenished using Vector. The expression of DDX3X and METTL3 were detected by western blot. (**B**) HEK293T cells were transfected with pHA-MT-A70 (0, 0.5, 1, and 2 μg, respectively) in 12-well plates. The expression of DDX3X and MT-A70 was detected by western blot. (**C**) Total RNA was extracted from HEK293T cells transfected with the vector, pMETTL3, or pMETTL3mut. The RNA expression of METTL3 was quantified via qRT-PCR. Data are presented as the means ± SEMs (n = 3). $^{**}P \leq 0.01$, unpaired Student's *t*-test. (**D**) pFlag-DDX3X and pHA-Ub were co-transfected with or without pMETTL3mut into HEK293T cells, followed by ubiquitination assay.

Table S1. shRNA and siRNA targeting sequences. Related to Figure 2 and Figure 5.

shRNA or siRNA	Sequence (5′ to 3′)
shMETTL3-1	CGTCAGTATCTTGGGCAAGTT
shMETTL3-2	GCCAAGGAACAATCCATTGTT
shDDX3X-1	CGGAGTGATTACGATGGCATT
shDDX3X-2	CGTAGAATAGTCGAACAAGAT
siDDX3X-1	CGAGAGAGTTGGCAGTACA
siDDX3X-2	CATTGAGCTTACTCGTTAT
sip65-1	GAUUGAGGAGAAACGUAAA
sip65-2	GAGGACAUUGAGGUGUAUU
siMDA5-1	GUCAUUAGUAAAUUUCGCA
siMDA5-2	GUUAUAGUUCUUGUCAAUA
siMettl3-1	GCACAUCCUACUCUUGUAA
siMettl3-2	GGACCAAGGAAGAGUGCAU

Table S2. Primers for RT-qPCR. Related to Figure 1, Figure 2, Figure 3, Figure 5, and Figure 6.

Gene	Species	Forward Primers (5'-3')	Reverse Primers (5'-3')
METTL3	Human/ Monkey	CAAGCTGCACTTCAGACGAA	GCTTGGCGTGTGGTCTTT
METTL3	Dog	CAAGCTGCACTTCAGACGAA	GCTAGGTGTATGGTCTTT
METTL3	Hamster	CAAGCTGCACTTCAGACGGA	GCTGGGCGTATGTTCCTT
DDX3X	Human	GGAGGAAGTACAGCCAGCAAAG	CTGCCAATGCCATCGTAATCACTC
EV71		CGAATGCTAGTGATGAGAGTAT	GAGGAAGATCTATCTCCCCAACT
GAPDH	Human	GAAGGTGAAGGTCGGAGTC	GAAGATGGTGATGGGATTTC
GAPDH	Monkey	CATCACTGCCACCCAGAAGACTG	ATGCCAGTGAGCTTCCCGTTCAG
GAPDH	Dog	GATGGTGAAGGTCGGAGTG	GAAGATGGAGATGGACTTCC
GAPDH	Hamster	CATCACGGCCACCCAGAAGACTG	ATGCCAGTGAGCTTCCCGTTCAG
IP10	Human	GGTGAGAAGAGATGTCTGAATCC	GTCCATCCTTGGAAGCACTGCA
IFNA	Human	AGAATCTCTCCTTTCTCCTG	TCTGACAACCTCCCAGGCAC
IFNB	Human	CTTGGATTCCTACAAAGAAGCAGC	TCCTCCTTCTGGAACTGCTGCA
RIG-I	Human	ACGCAGCCTGCAAGCCTTCC	TGTGGCAGCCTCCATTGGGC
ISG15	Human	AGGACAGGGTTCCCCTTGCC	CCTCCTGCCGGCTCACTTGC
ISG54	Human	CTGAACCGAGCCCTGCCGAAC	GCTGCCTCGTTTTGCCCTTTGAG
ISG56	Human	TCATCAGGTCAAGGATAGTC	CCACATTGTATTTGGTGTCTAGG
IL1B	Human	CCACAGACCTTCCAGGAGAATG	GTGCAGTTCAGTGATCGTACAGG
IL6	Human	GCCGCATCGCCGTCTCCTAC	CCTCAGCCCCCTCTGGGGTC
IL8	Human	GAGAGTGATTGAGAGTGGACCAC	CACAACCCTCTGCACCCAGTTT
IL18	Human	TCTTCATTGACCAAGGAAATCGG	TCCGGGGTGCATTATCTCTAC
Actb	Mouse	GGCTGTATTCCCCTCCATCG	CCAGTTGGTAACAATGCCATGT
Mettl3	Mouse	CTGGGCACTTGGATTTAAGGAA	TGAGAGGTGGTGTAGCAACTT
Ifnb	Mouse	TACAACAGCTACGCCTGGAT	AGTCCGCCTCTGATGCTTAA
Isg15	Mouse	GGTGTCCGTGACTAACTCCAT	TGGAAAGGGTAAGACCGTCCT
Isg56	Mouse	CTGAGATGTCACTTCACATGGAA	GTGCATCCCCAATGGGTTCT
